# Mapping the Hierarchical Environmental Transformations of Nanoscale UiO-66 Metal–organic Framework

**DOI:** 10.1021/acs.est.5c14487

**Published:** 2026-01-02

**Authors:** Swaroop Chakraborty, Iuliia Mikulska, Pankti Dhumal, Nathan Langford, Susan Nehzati, Rhiannon Boseley, Sang Pham, Christian Pfrang, Manpreet Kaur, Eugenia Valsami-Jones, Konstantin Ignatyev, Dhruv Menon, Superb K. Misra, Iseult Lynch

**Affiliations:** † School of Geography, Earth & Environmental Science, 1724 University of Birmingham, Edgbaston, Birmingham B15 2TT, U.K.; ‡ Centre for Environmental Research and Justice, University of Birmingham, Edgbaston, Birmingham B15 2TT, U.K.; § Diamond Light Source, 120796 Harwell Science and Innovation Campus, Didcot OX11 0DE, U.K.; ∥ Facility of Electron Microscopy, University of Birmingham, Edgbaston, Birmingham B15 2TT, U.K.; ⊥ Department of Chemical Engineering & Biotechnology, University of Cambridge, Cambridge CB3 0AS, U.K.; # Materials Engineering, 242275 Indian Institute of Technology, Palaj, Gandhinagar 382355, India

**Keywords:** metal organic frameworks (MOFs), safe-and-sustainable by design, hierarchical transformations, Daphnia magna, ecotoxicology

## Abstract

Metal–organic frameworks (MOFs) hold immense potential for applications from separations to catalysis, yet their long-term behavior across real-world environments remains unclear. Here we introduce a hierarchical exposure framework that tracks the structural and chemical transformations in the archetypal zirconium MOF UiO-66 across sequential compartments—atmospheric gases, air, aqueous media and a biological host—and resolves how prior exposures condition or prime subsequent transformations. Using synchrotron-based spectroscopy, we find that oxidative/reactive gases leave the Zr-carboxylate nodes essentially intact, whereas exposure to environmentally relevant aqueous media initiates partial shifts in local Zr coordination and introduces oxygen into the pores—with transformation extent governed by the chemistry of the environmental matrices. Strikingly, acute exposure (24 h) to the water flea *Daphnia magna* drives profound framework degradation and respeciation to Zr hydroxide species. Microfocus XRF maps show that Zr is highly localized in the animal’s digestive tract, and region-specific XANES confirms uniform speciation across its tissues. Our findings establish a paradigm shifting cross-compartment transformation hierarchy in which biological processes can dominate the fate of stable MOFs even when abiotic exposures appear benign. Thus, organism-level biotransformation should be performed as a necessary part of environmental safety assessments and materials design.

## Graphical abstract

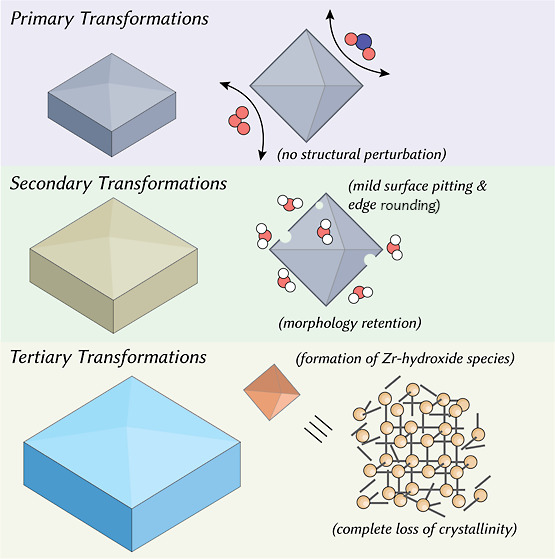

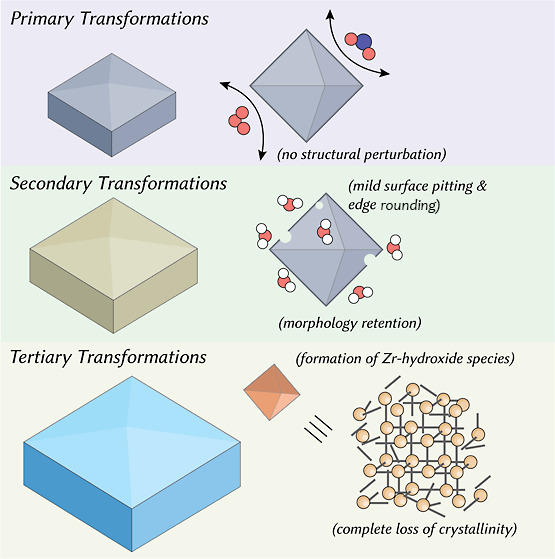

## 1 Significance

Metal–organic frameworks (MOFs) are deployed for separations, catalysis, sensing, and drug delivery, yet robust, predictive assessments of their environmental fate are lacking. We establish a generalizable transformation-hierarchy paradigm that tracks a material’s sequential aging across atmospheric, aqueous, and biotic compartments and quantifies how prior exposures condition subsequent chemistry. Using UiO-66 as an archetype, synchrotron X-ray absorption spectroscopy and whole-organism assays show minimal abiotic alteration but rapid in vivo respeciation in *Daphnia magna* to disordered Zr hydroxide species, demonstrating that biological processes can dominate ultimate outcomes. This integrative protocol provides a transferable basis to stress-test, rank, and model MOFs and other advanced materials, enabling mechanism-anchored predictions, safer-by-design optimization, and evidence-based regulation across applications from water treatment and gas separations to therapeutics.

## 2 Introduction

Metal–organic frameworks (MOFs) have emerged as a versatile class of porous materials known for their exceptionally high surface areas (often >1000 m^2^ g^–1^) and tunable chemistry. These attributes underpin a broad range of potential applications, from gas adsorption^1^ and separation to catalysis,^2^ drug delivery,^3^ and pollutant removal.^4^ Among MOFs, the zirconium-based UiO-66 family (UiO = University of Oslo who were the first to report them) is frequently highlighted for its robust structure and chemical versatility. The strong Zr–O coordination bonds in UiO-66 confer higher kinetic stability compared to many other MOFs,^5^ making this material an archetypal system in both fundamental research and applied technologies. Indeed, the combination of stability and functionality of UiO-66 has positioned it as a promising platform for real-world use in harsh conditions where many MOFs would otherwise deteriorate.

Although UiO-66 is one of the most intensively studied MOFs due to its exceptional stability, it has yet to see medium- or large-scale industrial deployment; most MOFs in-fact remain at the precommercial or early use stage.^6^ This makes robust, life-cycle-relevant transformation data essential for safe and sustainable translation.^7,8^ Despite UiO-66’s reputation for stability, questions remain about its transformation under complex environmental conditions. MOFs in general are less stable than traditional inorganic porous materials (e.g., zeolites), and even “stable” frameworks like UiO-66 can undergo gradual transformation when exposed to challenging environments. For instance, in the atmosphere, oxidizing pollutants such as nitrogen dioxide (NO_2_) and ozone (O_3_) can chemically attack MOFs. NO_2_ in humid air will form nitric and nitrous acids (HNO_3_/HNO_2_) that can protonate organic linkers, leading to linker bond breakage and framework collapse.^9^ Highly reactive oxidative species like NO^+^ can directly react with aromatic linkers or metal nodes, causing irreversible damage to the MOF’s coordination framework.^9,10^ Likewise, O_3_—a strong oxidant—is expected to oxidize organic linkers or metal clusters, initiating structural breakdown. In aqueous environments, UiO-66 could face further challenges. Water can hydrolyze the metal–ligand bonds, especially under non-neutral pH or in the presence of competing ions and organic matter. Prior studies have shown that while UiO-66 is quite stable in pure neutral water, it still releases small amounts of its terephthalate linkers over time; at moderately high pH (e.g., ∼7–8), linker leaching accelerates, and at even more basic pH the framework can completely decompose into Zr–oxide and free ligands.^11,12^ Such transformations—including partial dissolution, ligand exchange, or structural rearrangements—inevitably compromise the material’s performance (e.g., loss of surface area and adsorption capacity) and could pose environmental or health risks if toxic decomposition products or metal ions are released.^13^

A key knowledge gap lies in elucidating the sequential, hierarchical progression of these transformations.^14^ To the best of our knowledge, no single study has systematically captured the full continuum—from initial atmospheric exposure (air and/or reactive gases), through abiotic aqueous conditions, to biotic environments, within one integrated investigation.^15^ Real-world scenarios may involve multistage exposures: a MOF initially exposed to polluted air might be chemically “primed” by oxidation before it encounters water or biological systems. We hypothesize that an initial atmospheric exposure could create microstructural defects or reactive moieties in UiO-66, which in turn make it more susceptible to faster breakdown in water; subsequent aquatic dissolution or reorganization could then influence the material’s interactions and effects in living organisms. However, this cascade of environmental aging—from primary (atmospheric) to secondary (aquatic) to tertiary (biotic) transformations has not been thoroughly investigated for MOFs.^15^ Most stability studies to date have examined individual stressors in isolation, and thus the compounded impact of sequential exposures remains poorly understood or entirely missing.^16,17^

Here, we address this gap by mapping the transformation pathways of UiO-66 across environmental compartments.^14^ Using advanced synchrotron-based X-ray absorption spectroscopy techniques, we track chemical state changes and structural integrity of UiO-66 after simulated atmospheric oxidation and then after prolonged aquatic immersion.^18^ We further introduce the “aged” MOFs into an in vivo context using *D. magna*, a freshwater aquatic organism and model species in ecotoxicology, to probe biotic interactions and potential toxicological outcomes.^19^ *D. magna* is a sensitive indicator of waterborne pollutants and is widely employed in nanomaterial safety assessments, making it an ideal organism to evaluate any adverse effects of UiO-66 and its transformation products. Synchrotron-based X-ray absorption spectroscopy (XAS) and microfocus X-ray fluorescence (XRF) were critical for resolving the subtle chemical and structural changes in UiO-66 that occur during environmental and biotic transformations. XAS provided high sensitivity to detect minor shifts in coordination environment and bond distances, while microfocus XRF mapping allowed spatially resolved identification of zirconium speciation within daphnid tissues, revealing transformation patterns that would be masked in bulk measurements. By integrating these approaches, our study follows UiO-66 through a ‘life-cycle’ inspired sequence of environments, providing a comprehensive view of how its structure and properties evolve from air to water to biological exposure.

Finally, we discuss how understanding these sequential degradation processes can inform better design and deployment of MOFs. Insights from our findings point to design strategies (e.g., linker functionalization, node modifications, or coatings) that could enhance the MOF’s resilience against oxidative or hydrolytic attack, thereby preserving functionality. More broadly, this work aligns with the emerging “safe and sustainable by design” (SSbD) paradigm for advanced materials.^15,20,21^ The SSbD framework advocates for integrating safety and sustainability considerations *proactively* in material development, evaluating material performance *alongside* its environmental impact at all life-cycle stages (manufacture, formulation, use, disposal). By illuminating UiO-66’s degradation pathways and potential risks, we aim to guide the development of MOFs that maintain their desirable properties while minimizing environmental health risks, in essence, advancing UiO-66 and related materials toward an ethos of safe and sustainable design. Such an approach ensures that novel MOF-based technologies can be adopted with confidence in their long-term stability and environmental compatibility, ultimately supporting their sustainable deployment in real-world applications.

## 3 Results and Discussion

### 3.1 MOF Characterization

The morphology of as-synthesized UiO-66 was first examined by scanning electron microscopy (SEM; Figure S1a), revealing uniform, well-faceted octahedral crystals with smooth surfaces. Transmission electron microscopy (TEM; Figure S1b) confirmed the polyhedral morphology and provided further evidence of a narrow size distribution, with an average particle size of ∼66 nm (Figure S1b inset). The absence of amorphous surface layers is consistent with the high crystallinity expected for well-formed nanoscale UiO-66. Powder X-ray diffraction (PXRD; Figure S1c) confirmed the crystalline phase and structural purity. The diffraction pattern displayed intense reflections at 2θ ≈ 7.4°, 8.5°, and 25.8°, characteristic of the face-centered cubic topology of UiO-66. The close match with the simulated pattern and absence of additional peaks confirmed the formation of a single crystalline phase without detectable impurities.

XAS is powerful spectroscopic technique that is used to study the electronic properties and atomic local structure of materials. XAS spectra consist of X-ray absorption near edge structure (XANES) and extended X-ray absorption fine structure (EXAFS) regions. The XANES region contains information about electronic structure, oxidation state and coordination geometry of the specific element in the material. The EXAFS region of the XAS spectrum reveals details about the local atomic structure surrounding the absorbing atom. The Zr K-edge XANES spectra of the activated UiO-66 were compared with that of a Zr oxide (ZrO_2_), Zr hydroxide (Zr­(OH)_4_), Zr chloride (ZrCl_4_) and Zr hydrogen phosphate (Zr­(HPO_4_)_2_) reference compounds, which contains zirconium exclusively in the +4 oxidation state (Figure S5). The energy position of the absorption edge in the UiO-66 sample closely matches that of the reference compounds, indicating that the electronic environment around the Zr centers in UiO-66 corresponds to oxidation state of Zr^4+^. The Zr K-edge XANES spectra of as-synthesized and activated UiO-66 are similar, with only slight variations in the white line intensity: the white line of the activated spectrum is higher than that of the as-synthesized (Figure S6a). This increase in white line intensity after removing guest molecules from the pores of UiO-66 may be linked to minor distortions in the local structure around Zr atoms. These changes are also supported by a qualitative comparison of the EXAFS spectra, where the amplitude of oscillations increases after sample activation (Figure S6b). The distortions in the local structure around Zr atoms are more evident when comparing the Fourier Transform (FT) magnitudes of the EXAFS spectra (Figure S6c). The shape of the FT magnitudes obtained in this study closely resembles those reported by Ronda-Lloret and co-workers for activated UiO-66.^22^ Three distinct coordination shells are observed in the FT magnitudes. The first peak (at 1.54 Å) and the second peak (at 1.87 Å) correspond to the contributions of bridging oxygen atoms and carboxylate groups, respectively. The third peak (at 3.17 Å) could be associated with contributions from C and Zr atoms. After the activation process, the intensity of the second and third peaks increases. The changes in the XANES and EXAFS regions upon activation indicate distortions in the local structure around Zr atoms, associated with the removal of guest molecules from the pores of the MOF. These results were also confirmed by quantitative EXAFS data analysis (Tables S2 and S3). Valenzano and co-workers demonstrated similar results when the EXAFS spectrum of the UiO-66 was significantly affected by activation procedures.^23^ This combined morphological, crystallographic, and atomistic characterization confirms the high quality of the synthesized UiO-66, thereby providing a robust baseline for subsequent environmental and biotic transformation studies.

### 3.2 Primary Transformations of UiO-66 in Air and Reactive Gases

UiO-66 exhibits remarkable resilience when exposed to oxidative gases, as evidenced by the minimal changes in its Zr K-edge XANES and EXAFS spectra after gas-phase treatments (Figure 1a,c). The XANES edge position (∼18014.4 eV) remains essentially unchanged for all gas exposures (including air, O_3_ up to 10 ppm, and NO_2_ up to 10 ppm), indicating that the Zr centers retain their +4 oxidation state (Figure 1a). Furthermore, the white-line intensity (the postedge peak in the XANES) varies by less than ∼5% even under the most aggressive conditions (e.g., 10 ppm of O_3_ for 2 h), suggesting only negligible alteration of the Zr coordination environment. In other words, strong oxidants like ozone and NO_2_ do not measurably oxidize Zr or remove its coordinating ligands in the dry state. This stability of UiO-66 can be attributed to the inherently robust Zr–carboxylate bonds and the 12-connected Zr_6_O_4_(OH)_4_ node of UiO-66.^12^ Indeed, tetravalent metal–ligand bonds (such as Zr–O carboxylates) are among the strongest in MOFs, underpinning the exceptional gas-phase stability of UiO-66. Consistent with this, we observe no edge energy shift in the XANES and only trivial changes in intensity, confirming that the valence state and electronic structure of Zr remains intact after gas exposure.

**Figure 1:**
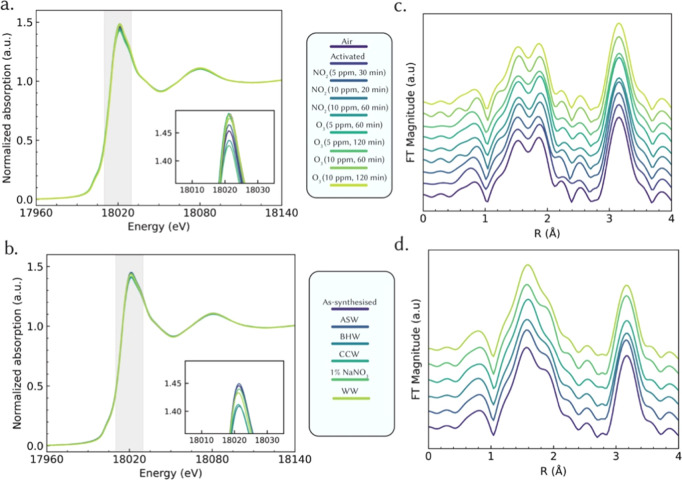
Zr K-edge XAS analysis of UiO-66 under atmospheric and environmental exposure conditions. (a) Normalized Zr K-edge XANES spectra showing the effect of gas-phase pollutants exposure (NO_2_ and O_3_) at varying concentrations and durations compared to air-exposed and thermally activated samples. The inset highlights subtle white line variations. (b) Normalized Zr K-edge XANES spectra for UiO-66 exposed to different aqueous media (1 mM NaNO_3_, ASW, WW, BHW, and CCM), compared to the as-synthesized UiO-66. (c) Fourier-transformed (FT) EXAFS spectra for activated UiO-66 sample and then exposed to gas-phase environments, revealing coordination environment changes relative to air and activated controls. (d) FT-EXAFS spectra for UiO-66 samples first exposed to air and subsequently to aqueous media. FT-EXAFS spectra are vertically offset for clarity. All FT-EXAFS spectra were obtained over the k-range of 3.6–15.2 Å^–1^ using k^3^-weighting.

EXAFS provides further evidence of UiO-66’s robustness under gas-phase transformation. The qualitative comparison of Fourier transform magnitude (Figure 1C) for O_3_ or NO_2_—exposed samples closely match those of the as-synthesized material. The first-shell peak in the Fourier-transformed EXAFS, centered at ∼1.7 Å (phase-uncorrected), corresponds to Zr–O bonds and remains at the same radial distance for all samples. Its amplitude is only slightly diminished (within ∼10% attenuation) after the harshest gas exposures, indicating a very modest increase in disorder or coordination. Likewise, the prominent second-shell feature around ∼3.3–3.5 Å—arising from Zr–Zr pairs in the hexanuclear node (and minor contributions from Zr–C paths through the organic linkers)—remains pronounced and at the same position in gas-exposed UiO-66. This confirms that the cluster connectivity and long-range structural order are largely preserved. Quantitative Zr K-edge EXAFS analysis indicates that samples exposed to 5 ppm of O_3_ retain the same local structure around Zr atoms as the activated sample (Table S3). In contrast, samples exposed to air, NO_2_, and 10 ppm of O_3_ show evidence of an additional oxygen or nitrogen atom at approximately 2.4 Å. Due to the limitations of EXAFS, it is not possible to distinguish between these atoms. This additional scattering path is likely attributed to a guest molecule, such as O_3_, H_2_O, or NO_2_ located within the pores of the UiO-66 framework. This finding is consistent with the reduction in XANES white line intensity, indicating minor alterations in the Zr electronic state, likely due to weak interactions with guest molecules within the UiO-66 pores rather than significant framework degradation. Bulk crystallinity after gas-phase aging was further assessed by PXRD on activated UiO-66 and samples exposed to air, 10 ppm of O_2_ and 5 ppm of NO_2_ (Figure S4). All patterns display the characteristic UiO-66 reflections with no additional peaks, confirming retention of the *fcu* framework under oxidative gas exposure; minor differences in relative peak intensities compared with Figure 2d reflect normal batch-to-batch variation rather than phase change.

**Figure 2:**
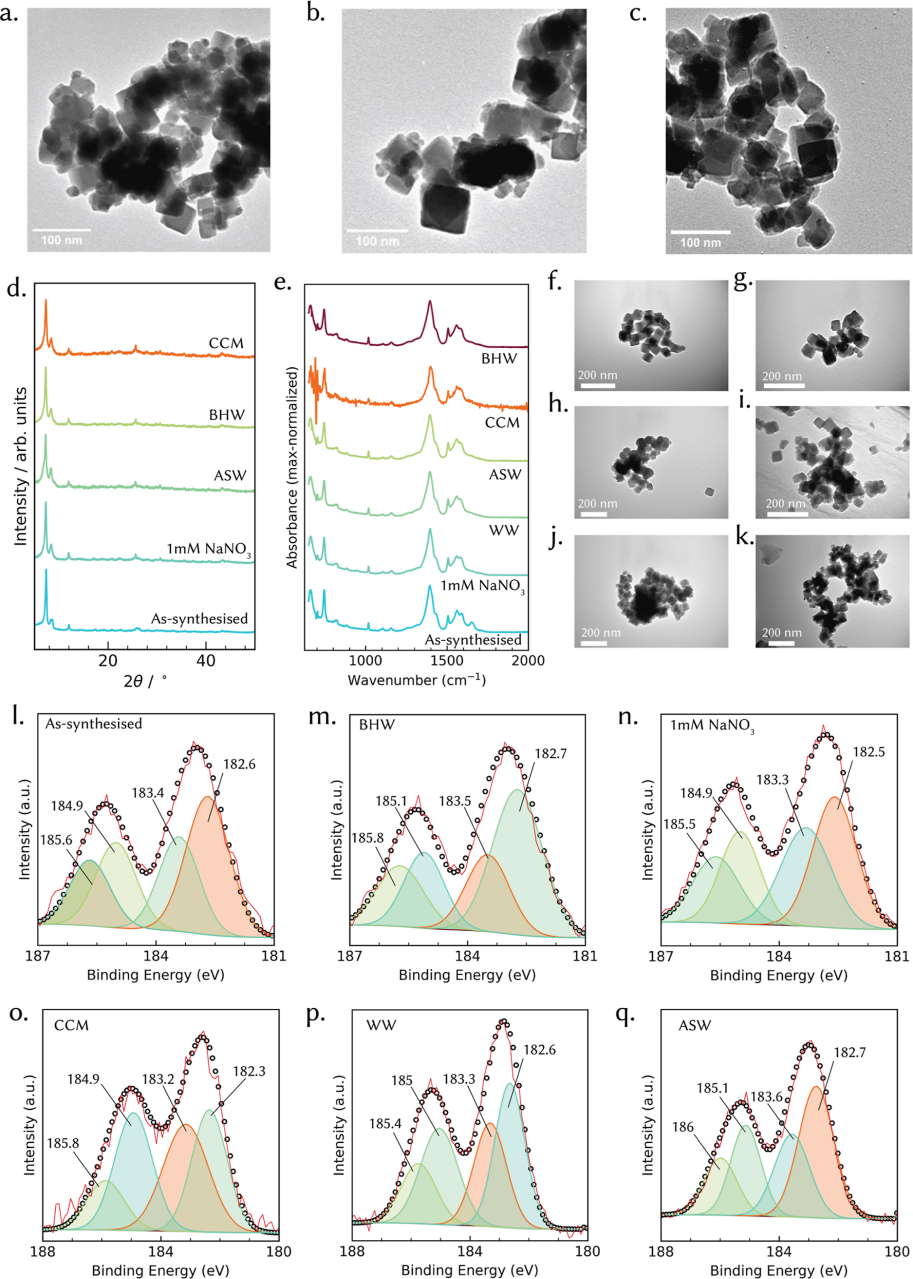
Multimodal characterization of UiO-66 following atmospheric and aqueous medium exposures. (a–c) TEM images of activated UiO-66 exposed to air (a), NO_2_ (**b**), and O_3_ (c), showing no significant morphological changes compared to activated UiO-66 material (f). (d) Comparative PXRD patterns of as-synthesized and medium-exposed UiO-66, highlighting retention of the primary crystalline framework with varying degrees of peak broadening. (e) FTIR spectra showing shifts and intensity changes in vibrational modes associated with linker coordination and surface hydroxyl groups. (f–k) TEM images of activated UiO-66 after exposure to different aqueous media, activated (f), BHW (g), ASW (h), CCM (i), WW (j), and 1 mM NaNO_3_ (k)—revealing preservation of polyhedral morphology. (l–q) High-resolution XPS spectra of the Zr 3d region for as-synthesized UiO-66 and medium-exposed samples (BHW, 1 mM NaNO_3_, CCM, WW, ASW), illustrating subtle shifts in binding energies and changes in Zr 3d_5/2_ and Zr 3d_3/2_ peak intensity ratios, consistent with alterations in Zr–O bonding environments and possible surface complexation or hydrolysis.

We note that prior studies have shown NO_2_ can break Zr–BDC bonds, forming Zr–nitrate species on the nodes.^24^ Thus, under dry oxidative conditions, UiO-66 undergoes only primary (very limited) transformation—a testament to its high chemical durability. The framework’s ability to withstand gas-phase oxidants without significant structural reorganization is an encouraging sign for applications in air pollution control or gas storage, as it suggests the material’s adsorptive sites and crystallinity will persist. However, stability alone does not preclude performance loss via pore-blocking deposits; unlike conventional catalysts, many MOFs cannot undergo high-temperature oxidative regeneration, and analogous fouling could reduce capacity in gas-purification streams containing condensable organics or particulates. In summary, the primary gas-phase transformations of UiO-66 are marginal, highlighting a resilience that stems from its robust coordination chemistry and structure. We note that our gas-phase experiment interrogates early stage stability under accelerated ppm-level conditions (30–120 min). Time-dependent transformations that could emerge under longer exposures (or under lower, environmentally realistic ppb levels over extended periods) were not assessed here and should be addressed in future time-resolved studies.

### 3.3 Secondary Aqueous-Phase Transformations of UiO-66

Similar to gas phase stability, exposure to aqueous environments demonstrates the robustness of the UiO-66 material. Figure 1b,d compares the Zr K-edge XANES and FT magnitudes of air exposed UiO-66 after immersion in various water-based media of increasing complexity: 1 mM NaNO_3_, Artificial Seawater (ASW), Borehole Water (BHW) from the University of Birmingham, Simulated Wastewater (WW), and nutrient-rich Cell Culture Media (CCM). Notably, all aqueous-treated samples show the Zr K-edge at 18014.4 keV with no significant energy shift relative to the as-synthesized material, confirming that Zr remains in the +4 state. However, subtle differences in the XANES white-line intensity are discernible among the media (Figure 1b). For instance, samples aged in complex media such as WW and CCM exhibit a slight decrease in white-line intensity (on the order of a few percent) compared to those in 1 mM NaNO_3_ and ASW. This decrease in white-line height suggests a minor reduction in the occupancy of coordinating ligands or an increased electron density at Zr, consistent with a small loss of carboxylate linkers and their partial replacement by hydroxide or water ligands. In simpler terms, the Zr coordination environment in BHW, WW, and CCM becomes marginally less saturated with organic linkers, reflecting the onset of hydrolysis or exchange reactions in these media.^11^ Importantly, these conclusions are based on Zr-centered speciation changes (XAS/XPS) and do not directly quantify linker release or organic byproducts. Ligand-resolved pathways (e.g., terephthalate liberation, linker modification, or impurity formation) therefore remain unresolved in the present study. By contrast, UiO-66 in 1 mM NaNO_3_ or ASW shows virtually unchanged XANES features, indicating that in the absence of natural organic matter or complex ions, the framework endures with minimal perturbation. These findings align with the known stability of UiO-66 in neutral water and the accelerating effect that dissolved ions and organics can have on MOF degradation.^5^ Even so, the changes in XANES features are slight, implying that aqueous exposure causes no gross change in Zr-oxidation state but rather subtle changes in electronic structure.

The quantitative EXAFS data (Figure S7b) provides deeper insight into the structural transformations of UiO-66 in water system. In all the samples exposed to liquid media we observed an additional O/N atom that is located in the pore of UiO-66. Crucially, despite these modifications, the absence of any new emergent peaks in the FT and the persistence of the core Zr–O and Zr–Zr features demonstrate that UiO-66’s underlying node structure survives aqueous exposure. To quantify the impact of sequential aging on porosity, N_2_ adsorption–desorption isotherms were collected for activated UiO-66 and for samples aged for 7 days in air, BHW and CCM (Figure S4). All samples exhibit the typical microporous UiO-66 isotherm shape, indicating that the underlying framework remains intact after these treatments. However, the BET surface area decreases from 1606 m^2^ g^–1^ for activated UiO-66 to 1149 m^2^ g^–1^ after air exposure, 1375 m^2^ g^–1^ after CCM exposure and 832 m^2^ g^–1^ after BHW exposure, with concomitant changes in total pore volume and apparent pore diameter (Table S4). The modest loss of surface area in air- and CCM-aged samples is consistent with minor surface carbonation or partial pore blocking, whereas the larger decrease after BHW exposure reflects more substantial pore blocking/etching. These textural data therefore support our conclusion that UiO-66 remains structurally robust under gas-phase and complex biological conditions, but experiences more pronounced porosity loss in simple electrolyte-rich freshwater.

TEM imaging of UiO-66 following air (Figure 2a), NO_2_ (Figure 2b) and O_3_ (Figure 2c) exposures shows well-defined polyhedral crystallites with sharp facets, closely resembling the as-synthesized material in size and overall morphology. Within the spatial resolution and contrast limits of TEM for these beam-sensitive particles, no clear evidence of particle fracture or morphological collapse is observed. We note that TEM provides a 2D projection of 3D particles and is not ideally suited to resolving very shallow surface roughening; any gas-induced surface modification, if present, is therefore expected to be subtle. These observations are consistent with the Zr K-edge EXAFS results (Figure 1c), which show minimal attenuation of the first-shell Zr–O and second-shell Zr–Zr coordination peaks, indicating that the primary node connectivity and inorganic coordination environment remain largely intact after gas-phase aging. The TEM and EXAFS data indicate that oxidative gas treatments induce only superficial changes at the particle surface, without measurable framework degradation at the atomic scale. Powder XRD patterns (Figure 2d) show that all exposed samples retain the characteristic UiO-66 reflections at ∼7.4° and ∼8.5° (indexed to the (111) and (200) planes), confirming no bulk amorphization. In 1 mM NaNO_3_ and ASW, peak positions and intensities closely match those of the as-synthesized sample, indicating high stability. In contrast, BHW, WW, and CCM samples show a modest reduction in peak intensity, suggesting partial loss of long-range order or defect generation without full framework collapse. FTIR (Figure 2e) supports this interpretation. All samples retain the asymmetric (∼1575 cm^–1^) and symmetric (∼1395 cm^–1^) carboxylate stretches characteristic of Zr–BDC coordination. However, samples in BHW, WW, and CCM exhibit slight broadening and intensity changes in these bands, consistent with partial linker exchange or coordination of anions and biomolecules present in complex media. Similarly, TEM images of UiO-66 exposed to aqueous media (Figure 2f–k) reveal that the framework retains its characteristic polyhedral morphology across all conditions. In simple media such as 1 mM NaNO_3_ (Figure 2k) and ASW (Figure 2h), particles remain sharply faceted with no visible surface roughening, consistent with EXAFS data (Figure 1d) showing negligible changes in Zr–O and Zr–Zr coordination shells. In contrast, samples aged in more complex media—BHW (Figure 2g), CCM (Figure 2i), and WW (Figure 2j)—display subtle rounding of edges, mild surface pitting, and occasional particle agglomeration. These morphological alterations align with EXAFS observations of slight attenuation in coordination peak amplitudes and minor additional O/N scatterers within the pores, indicative of partial hydrolysis or surface ligand exchange. Notably, no gross morphological collapse or fragmentation was observed, supporting the conclusion that aqueous exposure primarily induces early stage surface modifications while leaving the core inorganic node structure intact. In summary, aqueous-phase transformations of UiO-66 are subtle but detectable—the framework remains largely intact, exposure to harsher water chemistries leads to a slight reconfiguration of the coordination environment around neighboring oxygen atoms.

The findings are further corroborated by several additional characterization data. The subtle coordination changes were further supported by XPS analysis, which offered insight into the surface chemistry and bonding evolution (Figures 2l–q, and S2). Survey spectra of all UiO-66 samples revealed consistent presence of Zr, C, and O. Deconvolution of the C 1s region (Figure S2a–f) showed three principal peaks at 284.8 eV (C–C), 286.1 eV (C–O), and 288.9 eV (O–C=O), corresponding to the terephthalate linker environment. These remained relatively unchanged across all media, indicating retention of the organic ligand framework. In contrast, O 1s spectra (Figure S2g–l) revealed media-dependent variations. In activated UiO-66, three deconvoluted peaks were observed at 533.4 eV (O in O–C=O), 531.6 eV (Zr–OH), and 530.7 eV (Zr–O–Zr).^25,26^ These results are in agreement with the EXAFS findings (Tables S2 and S3). Following exposure to CCM and WW, the intensity of the Zr–OH component increased slightly (Figure 2o,p), suggesting surface hydrolysis or exchange of bridging ligands with hydroxyl or water groups. This shift was absent in the 1 mM NaNO_3_ and ASW samples (Figure 2n,q), which showed profiles nearly identical to the as-synthesized material. Zr 3d spectra for all samples exhibited two principal peaks at 182.9 and 185.3 eV, assigned to Zr 3d_5/2_ and Zr 3d_3/2_ of Zr­(IV), respectively.^27^ Notably, a minor shoulder corresponding to Zr­(III) was detected but remained consistent in intensity across all aquatic samples, implying that aqueous exposure did not alter the formal oxidation state of Zr or induce surface reduction. This further confirms the XANES findings (Figure S5) that Zr remains in a stable oxidative environment.^28^ Taken together, these data suggest that while UiO-66 maintains its structural and chemical identity in simple aqueous systems, exposure to biologically and chemically complex media induces modest hydrolysis and surface recoordination events. These changes, while subtle at the atomic scale, may represent early stage transformations that precondition the material for further degradation upon entry into biological systems.

### 3.4 Biotic and Tertiary Transformation in *D. magna*

The final tier of the hierarchy probes what happens to UiO-66 once it is ingested by an organism. Neonate *D. magna* were exposed for 24 h to UiO-66 that had first been aged for 7 days in BHW (100 μg mL^–1^), after which the animals were prepared for microfocus XAS. Micro-XRF maps collected at I18 (Diamond Light Source) show that Zr derived from UiO-66 is not distributed uniformly through the body but is strongly concentrated along the intestinal tract (Figure 3a,b). The most intense Zr signals (Regions 1–4 in Figure 3b) coincide with the gut lumen and adjacent epithelium, whereas little or no Zr is detected elsewhere, indicating negligible translocation into other tissues. This pattern mirrors earlier observations for metal-based nanoparticles in *D. magna*, where metals accumulate predominantly in the midgut and digestive appendages and are often associated with local tissue perturbation.^29^ Within the present context it identifies the gut, with its enzyme-rich, organic and pH-variable microenvironment, as the principal site of UiO-66 transformation.

**Figure 3:**
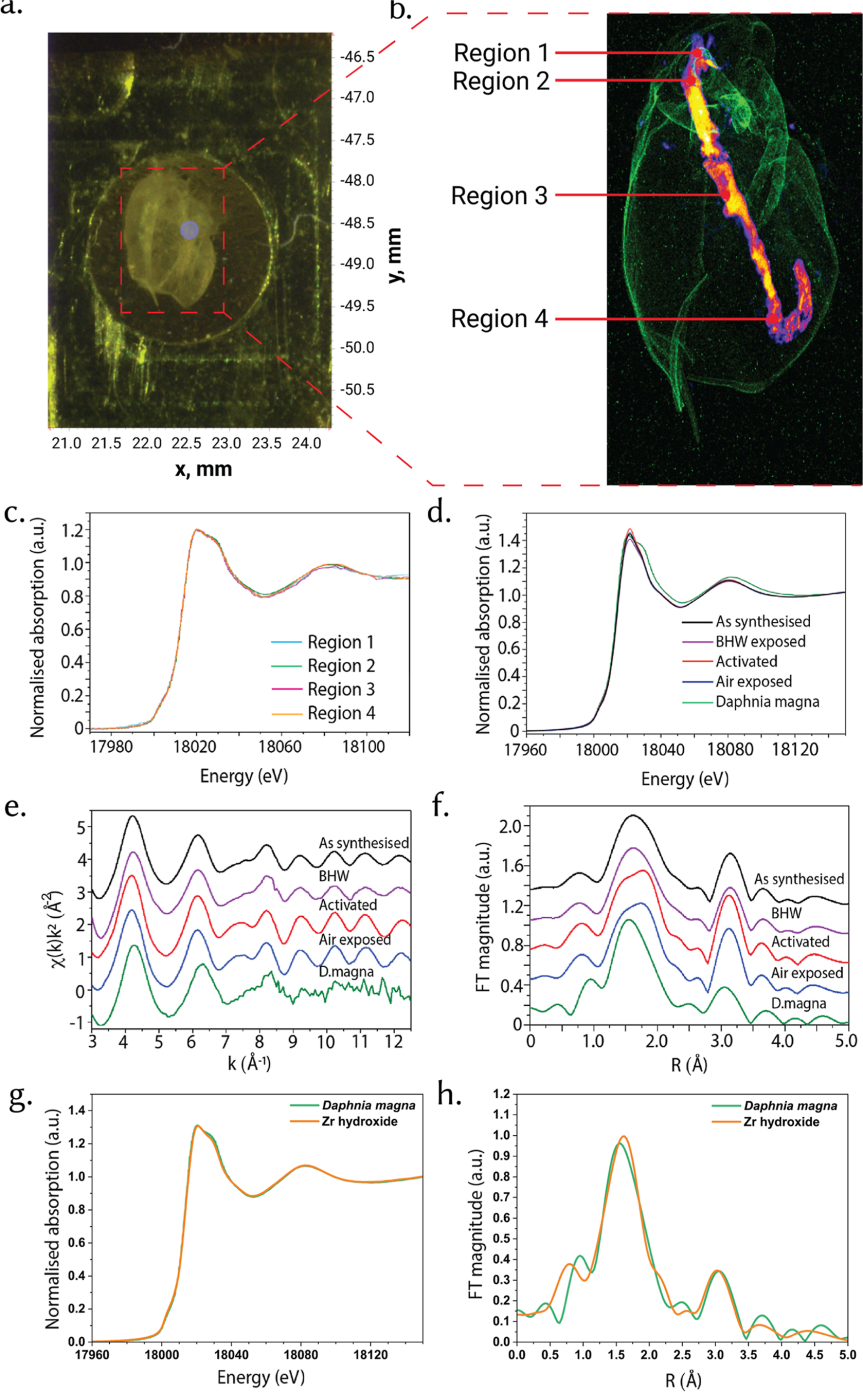
Biotic transformation of UiO-66 in *D. magna*visualized using synchrotron-based micro-XRF mapping and XAS. (a) Optical image of a *D. magna* specimen postexposure, mounted for μXRF and XAS analysis. (b) Elemental distribution map showing Zr localization within different anatomical regions (gut, appendages), with four distinct Region of Interest (ROIs) (Regions 1–4) identified for subsequent XAS analysis.^7^ [Color code: Green = Ca; Inferno = Zr localization] (c) Zr K-edge XANES spectra for individual ROIs within a daphnid, demonstrating minimal intraorganismal spectral variation. (d) Zr K-edge XANES comparison between as-synthesized UiO-66, abiotic controls (BHW exposed, air exposed, and activated UiO-66), and biotically transformed material recovered from *D. magna*. (e) Zr K-edge k^2^-weighted EXAFS spectra showing pronounced structural disorder in biotically transformed UiO-66 relative to abiotic controls. Spectra are shifted vertically for clarity. (f) Corresponding FT-EXAFS spectra indicating substantial reductions and shifts in Zr–O and Zr–Zr coordination shells for biotically transformed UiO-66, suggesting partial framework degradation and reprecipitation within the organism. FT-EXAFS spectra are vertically offset for clarity. (g) Comparison of Zr K-edge XANES profiles measured in *D. magna* and in Zr hydroxide reference compound. (h) Corresponding FT-EXAFS spectra highlighting subtle variations in the first and second coordination shells. All FT-EXAFS spectra were obtained over the k-range of 3.6–15.2 Å^–1^ using k^3^-weighting. Panel 3b and portions of panels 3c—f are reproduced from (ref 7). Copyright 2025 American Chemical Society.

Zr K-edge XANES spectra extracted from the four Zr-rich regions are virtually identical to one another but differ markedly from all abiotic UiO-66 references (as-synthesized, activated, air- and BHW-exposed; Figure 3c,d). The daphnid spectra exhibit a broader, less intense white line than the MOF standards, consistent with a more disordered and chemically altered Zr coordination environment. EXAFS analysis reinforces this—the Fourier-transformed |χ­(*R*)| of the daphnid sample shows a heavily damped first coordination shell and an almost complete loss of the second-shell feature that characterizes the ordered Zr_6_O_4_(OH)_4_—terephthalate node (Figure 3e,f). Such truncation of medium-range order is characteristic of amorphization or strong structural disorder; a similar collapse of higher-shell EXAFS features has been reported for mechanically milled nanocrystalline yttria-stabilized zirconia (YSZ), where local order persists only to the first shell.^30^ To identify the Zr species present in *D. magna*, we compared the Zr K-edge XANES and FT-EXAFS spectra with reference compounds, including Zr oxide (ZrO_2_), Zr hydroxide (Zr­(OH)_4_), Zr chloride (ZrCl_4_), and Zr hydrogen phosphate (Zr­(HPO_4_)_2_). The comparison shows that the Zr K-edge XANES profile closely matches that of Zr hydroxide, with only minor variations in the intensity of the spectral feature at 18,028 eV (Figure 3g). Similarly, qualitative analysis of FT magnitudes indicates that the local structure around Zr atoms is very similar to Zr hydroxide, with slight shifts in the positions of the first and second coordination shells (Figure 3h). These small differences observed in XANES and EXAFS spectra may be attributed to variations in measurement conditions across beamlines. The *D. magna* sample was analyzed at the I18 beamline using a Si(111) crystal cut of the monochromator and a microfocused beam, whereas reference compounds were measured at the B18 beamline using a Si(311) monochromator crystal cut and a 1 mm beam size. Comparable differences were noted when the same pellet was measured on both I18 and B18 beamlines (Figure S10). Furthermore, comparison of the Zr K-edge EXAFS spectra (not shown) for *D. magna* and the Zr hydroxide reference reveals an absence of oscillations at higher k-values, indicating that Zr hydroxide within the *D. magna* sample is more disordered than in the reference compound.

Taken together, the spatial localization of Zr to the gut, the uniform XANES signatures across different gut regions, and the loss of higher-shell EXAFS contributions all point to extensive in vivo respeciation of UiO-66 within the digestive tract. The simplest interpretation is that terephthalate linkers are displaced or degraded during digestion and the Zr nodes reprecipitate as poorly ordered Zr–O/OH (and likely Zr–phosphate) phases in a complex matrix of gut contents and microbiome.^31^ A more detailed mechanistic analysis of this biotic transformation, including its chronic reproductive consequences for *D. magna*, is provided in our recent companion study on UiO-66 in freshwater invertebrates.^7^ Aspects of the *D. magna* micro-XRF mapping and Zr K-edge EXAFS analysis (Figure 3b–f) were previously reported in the context of biotic transformation and ecotoxicological outcomes in the same source. Here, these in vivo results serve to anchor the upper level of the transformation hierarchy, demonstrating that a MOF that is robust to gas-phase and aqueous aging can nonetheless be rapidly “remade” inside an aquatic grazer into new, environmentally distinct Zr phases. The uniformity of the XANES across Regions 1–4 (Figure 3c) suggests that wherever Zr accumulates in the gut, it exists in the same transformed state. It is inferred that the MOF’s organic linkers (terephthalate) are released or replaced during digestion, and the Zr nodes are converted into new inorganic species. The absence of EXAFS oscillations beyond 9 Å^–1^, along with a marked reduction in FT EXAFS amplitudes and a shift in the first and second coordination shells (Figure 3h) in the daphnid sample, strongly indicates the formation of amorphous Zr hydroxide species.^32,33^ Therefore, when UiO-66 is introduced into an aquatic organism like *D. magna*, it does not remain as an intact framework; instead, it transforms in situ into a different Zr-bearing phase. This transformation, confined mainly to the digestive tract, highlights the importance of aquatic environmental conditions on MOF stability and has implications for the material’s fate and potential toxicity in real-world water systems and ecosystems.

TEM analysis of biotically transformed UiO-66 provides direct visual evidence of morphological evolution during the exposure–ingestion–excretion sequence in *D. magna* (Figure 4a–c). Following the hierarchical/sequential exposure, the air exposed UiO-66 (Figure 4a) exhibits sharply faceted octahedral crystals, characteristic of a defect-free, well-crystallized framework. After 7 days incubation in BHW prior to ingestion by daphnids, the particles (Figure 4b) retain their general polyhedral shape but display mild surface roughening, consistent with the modest attenuation of coordination shell intensities observed in EXAFS for BHW-aged samples (Figure 3f). Strikingly, UiO-66 recovered from *D. magna* faecal material postdepuration (Figure 4c) shows a complete loss of well-defined facets and the emergence of irregular, loosely agglomerated nanoscale domains. This morphological collapse mirrors the severe damping and near disappearance of the second-shell Zr–Zr feature in the corresponding EXAFS spectra, confirming that the original Zr_6_O_4_(OH)_4_ cluster connectivity has been disrupted. The TEM evidence therefore visually corroborates the spectroscopic finding that ingestion by daphnids induces a transition from a crystalline, faceted MOF to a disordered, likely amorphous Zr-bearing phase, formed via in-gut ligand loss, hydrolysis, and reprecipitation. In essence, the coordination environment of Zr is no longer that of the intact UiO-66 framework. This finding is further confirmed by selected area electron diffraction (SAED) of as-synthesized and postdepurated UiO-66 nanoparticles (Figure 4d,e) which clearly shows a crystalline to amorphous transition. Potentially, the MOF’s organic linkers may be partially displaced or replaced by biomolecules, or Zr may be complexed into new species (such as Zr–O, Zr–phosphate or Zr–protein complexes) in vivo. Scanning Transmission Electron Microscopy—High-Angle Annular Dark-Field (STEM-HAADF) imaging coupled with corresponding elemental maps further hint toward the formation of new species such as Zr-hydroxide (Figures 4f,g and S11a,b). The biotic processes in *D. magna* clearly disrupt the MOF’s structure to a far greater extent than any tested gas or aqueous exposure, highlighting the role of biotransformation in the fate of nanomaterials.

**Figure 4:**
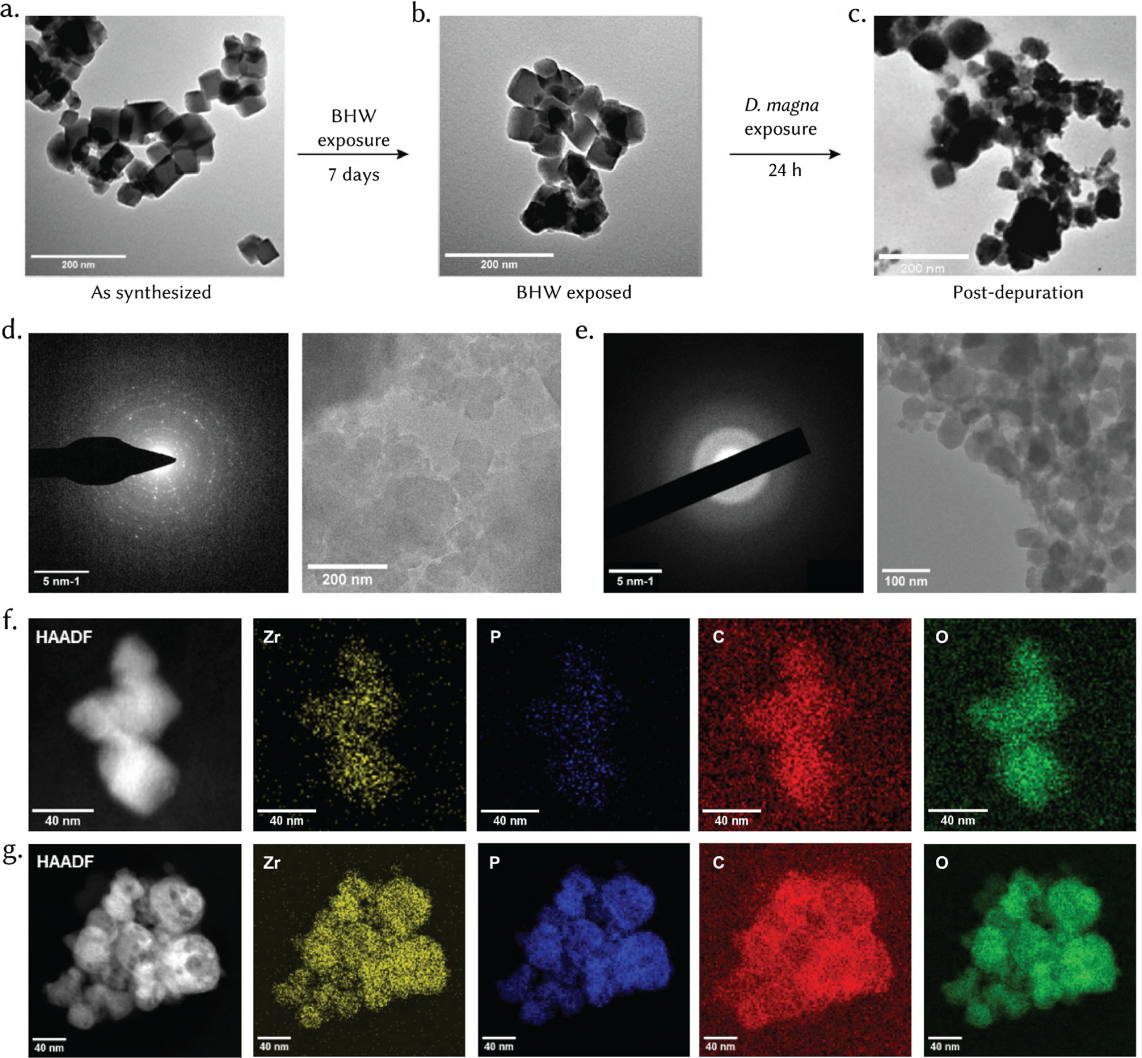
Postdepuration analysis of UiO-66 MOFs. (a) TEM image of air exposed UiO-66 nanoparticles showing well-defined polyhedral morphology and smooth surfaces, indicative of high crystallinity. (b) TEM image of UiO-66 after 7 days exposure to BHW, revealing aggregation and partial surface roughening consistent with initial structural degradation. (c) TEM image of UiO-66 particles excreted by *D. magna* postdepuration, displaying irregular morphology and loss of distinct crystal facets, indicative of advanced structural transformation and amorphization. (d,e) Low-dose SAED and corresponding TEM image of as-synthesized UiO-66 nanoparticles (d) and postdepurated UiO-66 particles (e) highlighting loss of crystallinity postbiotic exposure. (f,g) STEM HAADF images and EDS mapping for as-synthesized UiO-66 (f) and postdepurated UiO-66 (g).

To exclude the possibility that sample preparation artifacts (e.g., freezing and drying) were responsible for the observed Zr respeciation, UiO-66 was also exposed to BHW for 7 days and then subjected to the same freeze-drying protocol used for the *D. magna* samples. The resulting powder XRD pattern is indistinguishable from that of activated UiO-66 (Figure S12), confirming that our preparation procedure does not, by itself, induce framework collapse or formation of new crystalline Zr phases. In contrast, the micro-XAS spectra collected from *D. magna* guts remain clearly distinct from both pristine and BHW-only controls (Figure 3d–f), supporting the interpretation that the strong Zr–OH respeciation arises from the organismal microenvironment rather than ex situ sample handling.

## 4 Implications for Environmental Stability and Downstream Reactivity

Our findings clearly demonstrate that UiO-66 is exceptionally robust in air and reactive gas-phase and complex aquatic environmental conditions. This study demonstrates that biotic and environmental transformations of UiO-66 can be resolved at the atomistic scale, revealing changes that remain invisible to bulk analytical methods (Figure 5). Synchrotron-based Zr K-edge XAS and microfocus X-ray fluorescence mapping were instrumental in achieving this, as they provide the sensitivity and spatial resolution necessary to detect minute alterations in Zr coordination environments and linker integrity within complex biological matrices such as *D. magna* tissues. These capabilities allow us to directly link atomistic-level transformations to observed macroscopic effects, bridging the gap between structural changes and ecological outcomes (Figure 5).

**Figure 5:**
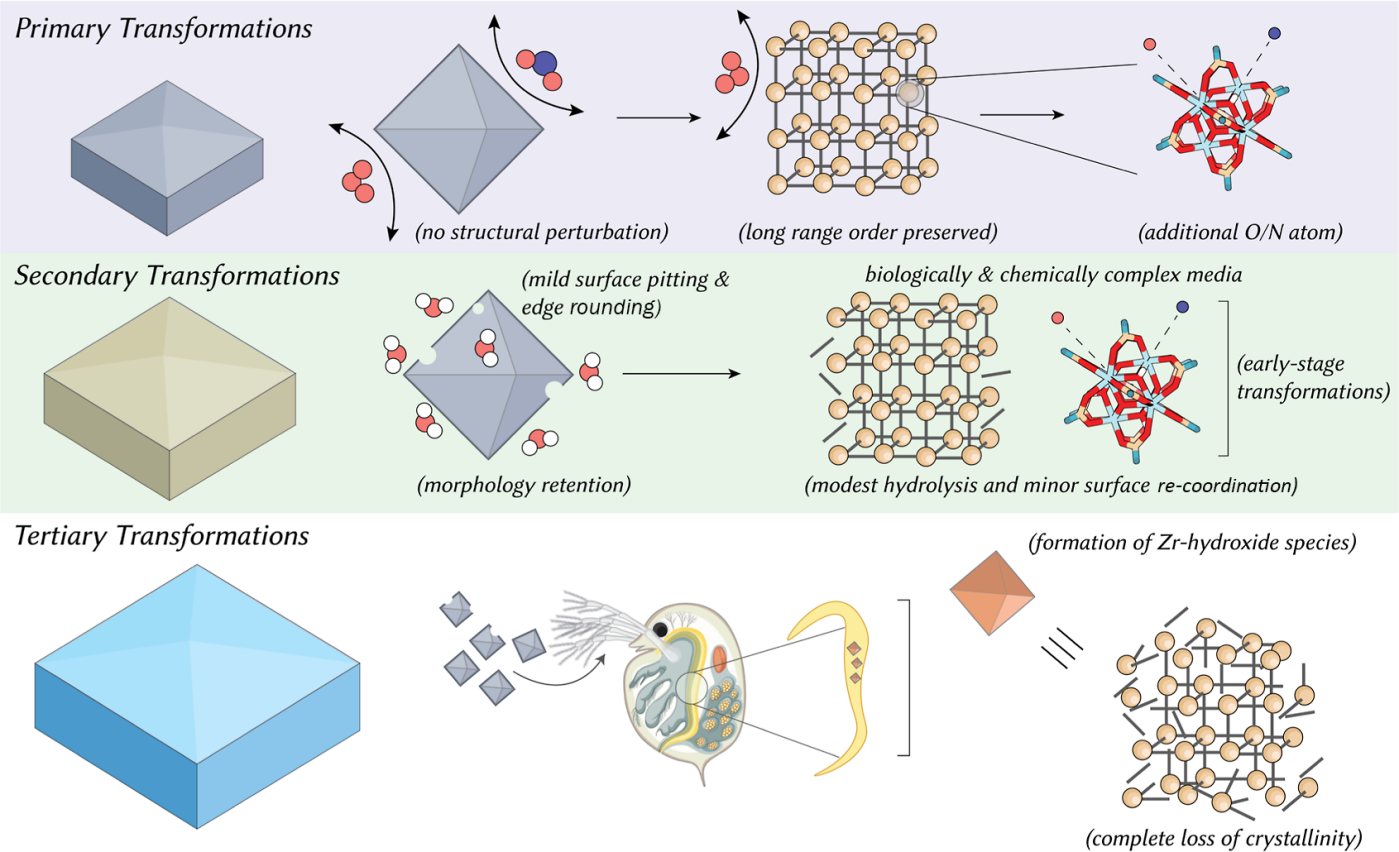
The transformation hierarchy. UiO-66 remains stable in harsh gas-phase, air and aqueous environments, but synchrotron Zr K-edge XAS and microfocus XRF reveal that biotic exposure in *D. magna* drives complete framework degradation and respeciation. Atomistic-scale analysis correlates subtle coordination changes and defect formation to complete organism-level transformation, highlighting that biological processes dominate MOF fate even when abiotic exposures appear relatively benign. These findings emphasize the need for hierarchical life-cycle inspired studies to capture realistic transformation pathways in MOF materials.

From a biological perspective, the subtle degradation observed in complex media (like nutrient-rich cell culture medium, CCM) indicates that when UiO-66 enters living systems (for example, in drug delivery or biomedical uses), it does not remain inert (Figure 5). The formation of missing-linker defects and the replacement of linkers by OH/H_2_O or other groups alters the surface chemistry—increasing the likelihood of releasing inorganic Zr species or organic linker fragments into the surroundings. In fact, in a whole-organism exposure using a model aquatic invertebrate (*D. magna*), we observed clear evidence of biotic transformation of UiO-66 (Figure 5). Taken together, our hierarchy indicates that once UiO-66 enters the biosphere it is likely to be respeciated by organismal microenvironments into amorphous, Zr-rich phases (e.g., hydroxide species as shown in Figure 3g,h) that can persist in the gut and be exported in dense faecal pellets, thereby promoting transfer from pelagic waters to sediments. Crucially, this biotic fate carried clear ecological cost: despite negligible abiotic dissolution and low acute toxicity, in vivo transformation coincided with strong, population-relevant reproductive impairment in *D. magna*, emphasizing that ‘stable’ MOFs may still generate hazard through biological remaking and redistribution.^7^ Qualitative comparison of the Fourier-transformed EXAFS magnitudes for MOF-exposed to daphnids and the as-synthesized/activated/air exposed UiO-66 sample reveals pronounced alterations in the first and second coordination shells. These changes indicate that UiO-66 undergoes significant structural transformations within daphnid gut. Simultaneously, synchrotron XRF mapping detected Zr from UiO-66 inside the gut of the organism, confirming that the MOF (or its components) is internalized and partially broken down in vivo. Such changes to UiO-66’s surface and structure likely affect how organisms interact with and respond to the material. For example, a higher density of –OH groups on the defected UiO-66 improves its dispersibility in water, but it also facilitates coordination of biomolecules to the MOF’s metal sites, potentially modifying the material’s bioavailability or toxicity profile. Likewise, gradual linker dissociation results in low-level leaching of terephthalate into solution, which must be considered for its downstream biological effects (e.g., metabolisation or signaling interference in organisms). A key limitation is that we could not fully resolve ligand-centric degradation pathways in these complex matrices. FTIR provides only qualitative sensitivity against strong media backgrounds, and digestion NMR is impractical for the *D. magna*/biotic samples in this format. Definitive mapping of linker fate will require complementary organic-analytical workflows (e.g., targeted LC–MS, digestion/solution NMR in simplified media, and/or isotopically labeled linkers).

In essence, these aqueous-phase transformations highlight a trade-off between stability and reactivity for UiO-66: the framework is stable enough to persist and function under realistic aqueous conditions, yet it is not so inert as to avoid all chemical change. This behavior aligns with UiO-66’s status as a *metastable* MOF under strongly oxidative or aquatic conditions—it resists outright disintegration but adapts by reconfiguring its coordination landscape (for instance, by losing linkers and attaching new ligands like –OH or inorganic anions).^12^ This adaptability can be either advantageous or detrimental depending on the context, and it highlights the importance of monitoring MOF integrity in any environmental or biological application. Contrary to our initial hypothesis (i.e., an initial atmospheric exposure could create microstructural defects or reactive moieties in UiO-66, which in turn make it more susceptible to faster breakdown in water; subsequent aquatic dissolution or reorganization could then influence the material’s interactions and effects in living organism), the results show that UiO-66’s crystalline network endures dry atmospheres and simple aqueous milieus. Instead, its major structural disruption occurred only upon biological exposure, where in vivo processes within *D. magna* rapidly (in <24 h) converted the framework into disordered Zr species. This implies that for highly robust MOFs like UiO-66, atmospheric or aqueous transformations alone are insufficient predictors of environmental fate, and that biotic processes dominate their ultimate breakdown. More broadly, it demonstrates the necessity of including whole-organism studies within hierarchical transformation assessments to fully capture realistic pathways of MOF transformation and risk. Thus, we establish a clear transformation hierarchy for UiO-66: resistant to atmospheric and aqueous challenges, but vulnerable to biotic reprocessing. This hierarchy must be integrated into future SSbD assessments. Further, future work will systematically test the applicability of the hierarchical transformation framework across diverse MOF families, using coordinated gas-phase, aqueous, and in vivo exposure sequences to quantify stability, transformation kinetics, and speciation outcomes.

From a SSbD perspective, several established stabilization levers could be evaluated within this hierarchy: increasing hydrophobicity via linker/surface modification or polymer shells to slow aquatic/biotic attack, linker functionalization or fluorinated/strengthened Zr nodes to resist ligand exchange, and defect-density control to minimize vulnerable missing-linker sites. These strategies provide clear, testable routes to shift the failure window observed here. Further detailed sequential transformation studies would be required using X-ray spectroscopic techniques to establish a clarity on UiO-66 stability in complex abiotic and biotic environmental conditions.^34–37^

## 5 Materials and Methods

### 5.1 Materials

All gases and reagents were of analytical grade. Nitrogen dioxide (NO_2_) and ozone (O_3_) gas mixtures were used for gas-phase exposure experiments at controlled parts-per-million (10 and 5 ppm) levels with nitrogen as a carrier gas. Five aqueous media were prepared to simulate various environmental and biological conditions: 1 mM NaNO_3_ solution, artificial seawater (ASW), simulated wastewater (WW), natural borehole water (BHW), and a nutrient-rich cell culture medium (CCM). The detailed composition is discussed in the Supporting Information file (Table S1). Biological Organism: The freshwater crustacean *D. magna* (a standard model organism in ecotoxicology, BHAM-2 strain) was used for biotic exposure studies.

### 5.2 Synthesis and Characterization of Nano-UiO-66 MOFs

Nanosized UiO-66 was synthesized via a solvothermal method. Zirconium chloride (ZrCl_4_; 0.6435 mmol, 0.14996 g) and benzene-1,4-dicarboxylic acid (BDC; 0.6435 mmol, 0.1069 g) were dissolved in 75 mL of *N*,*N*-Dimethylformamide (DMF) under continuous stirring for 30 min to ensure complete dissolution and precursor homogeneity. The clear solution was transferred to a Teflon-lined autoclave and heated at 120 °C for 24 h. After cooling to room temperature, the resulting precipitate was washed thoroughly with ultrapure water to remove unreacted precursors and residual solvent. The precipitate was then dried at 60 °C in the oven followed by vacuum-dried overnight at 120 °C for activation. No monocarboxylic acid or mineral-acid modulator was used in this synthesis. Now, activated samples were stored in a glovebox for reactive gases and air exposure for primary transformation. The preactivation UiO-66 has been referred as “as-synthesized” UiO-66.

The as-synthesized and activated UiO-66 powder were subjected to comprehensive structural and morphological characterization. Phase identification was performed by X-ray diffraction (XRD; Malvern Panalytical, Zr–Kα source, 40 kV, 30 mA, 2θ range: 5°–50°). Particle morphology and size distribution were examined by SEM (JEOL JSM-7900F) and TEM (JEOL 1400, operated at 80 kV electron voltage). Zirconium K-edge XAS measurements were conducted on as-synthesized, activated and exposed to various gaseous and aqueous media UiO-66 samples to identify the local geometry of the material at the B18 Core XAS beamline^38,39^ of the Diamond Light Source (experiment IDs: MG33674-1, SP35117-1, SP35776-1).^26,27^ Pt-coated collimating and focusing mirrors were employed, with an Si(111) double-crystal monochromator used to select the incident X-ray energy. A zirconium foil standard was measured simultaneously for energy calibration. The Zr K-edge XAS measurements on Zr oxide (ZrO_2_), Zr hydroxide (Zr­(OH)_4_), Zr chloride (ZrCl_4_), and Zr hydrogen phosphate (Zr­(HPO_4_)_2_) reference compounds were performed at B18 (experiment ID: SP40080-2) beamline using Pt-coated collimating and focusing mirrors with an Si(311) double-crystal monochromator. All measurements were performed in Quick EXAFS (QEXAFS) mode and in transmission geometry at room temperature. Sample preparation for XAS measurements varied depending on the environmental conditions to which the samples were exposed. Detailed procedures are provided in the respective sections below. Each sample was scanned three times to improve the signal-to-noise ratio and verify spectral reproducibility. Data reduction and analysis were carried out using the Demeter software package.^40^ Biotic micro-XRF and micro-XAS measurements on D. magna were carried out at the I18 microfocus beamline^39,41^ (Diamond Light Source, experiment ID SP40942-1). Lyophilized UiO-66—exposed daphnids mounted on Kapton tape were interrogated with a ∼2 μm hard X-ray beam tuned to 18.5 keV. Raster scans generated 2D XRF maps that resolved the distribution of Zr and major endogenous elements (e.g., Ca) within individual organisms. Zr K-edge XANES/EXAFS spectra were then acquired from regions of interest identified from these maps. A Si(111) double-crystal monochromator (similar to B18 beamline) was used for energy selection, with a Zr foil for energy calibration, and fluorescence was collected using multielement silicon drift detectors at 90° to the incident beam on the sample.^38^ Micro-XAS data sets were processed with the Demeter package in the same way as the bulk XAS, and XRF spectra and maps were fitted and quantified using PyMCA.^40^

### 5.3 Hierarchical Transformation Studies

#### 5.3.1 Primary Transformation (Air and Reactive Gases)

Before gas exposure, activated UiO-66 samples (100 mg) were placed in a round-bottom flask and kept under a flowing N_2_ atmosphere. All sample transfers and preparations were done in a nitrogen-filled glovebox or under flowing N_2_ to maintain an inert environment. The activated UiO-66 in the flask was exposed to controlled low concentrations of reactive gases to evaluate structural and chemical stability. NO_2_ and O_3_ were introduced (separately) at concentrations (mixing ratios) of 5 and 10 ppm for exposure durations of 30 min, 60 or 120 min. The reason for exceeding environmentally realistic level of O_3_ and NO_2_ was to investigate if UiO-66 could withstand such a high concentration of reactive gases without undergoing structural or chemical transformation. These exposures were designed as accelerated, short-duration stress tests to probe early gas–MOF interactions. Longer exposure durations at the same ppm conditions were not explored in this study, and time-dependent effects beyond 120 min therefore remain unresolved. During these exposures, the MOF powder was spread evenly throughout the vessel to obtain an even exposure to the gases. After the specified exposure time, the flask was purged with N_2_ and the sample was immediately transferred into an airtight container under inert atmosphere. In a glovebox, the exposed MOF powder was loaded into a 6 mm-diameter Kapton washer (170 μm thickness) and sealed with Kapton tape. The sealed washer was mounted on a sample holder that was then enclosed in an aluminized Mylar bag under N_2_ to prevent any contact with ambient air during storage, transport and XAS measurements. Gas-exposed samples, as well as an air-exposed control sample, were analyzed to identify any gas-induced transformations. The preserved samples were transported to Diamond Light Source for Zr K-edge X-ray absorption spectroscopy measurement at B18 beamline (details in Synchrotron based XAS Measurements above). In addition, aliquots of each sample were characterized by powder XRD, FTIR, and TEM, to probe changes in crystallinity, functional groups, and morphology caused by NO_2_ or O_3_ exposure, respectively.

#### 5.3.2 Secondary Transformation (Biological and Environmental Media)

The UiO-66 samples (pre-exposed to reactive gases and air) was subjected to aging in various aqueous media to simulate environmental and biological fluid conditions. Approximately 100 mg of air-exposed UiO-66 was dispersed in 100 mL of each test solution (100 mg/L concentration) and the mixtures were gently stirred for 7 days. Five different aqueous media were tested, representing a range of chemistries: 1 mM NaNO_3_ (in deionized water), ASW, WW, BHW, and a nutrient-rich CCM (Table S1). The selected media span a gradient of chemical complexity, ionic strength, and organic content, enabling systematic evaluation of UiO-66’s stability under environmentally and biologically relevant conditions. Simple electrolyte solutions such as 1 mM NaNO_3_ provide a baseline for assessing intrinsic hydrolytic resistance, while complex matrices like ASW and WW introduce multivalent ions and organic matter that can accelerate linker displacement or promote particle aggregation. Natural BHW offers a realistic freshwater exposure scenario, and nutrient-rich CCM mimics in vitro biological environments where strong biocorona formation and biotic transformations may occur. These contrasts in composition and pH span the distinct transformation pathways observed in subsequent aqueous-phase stability experiments. The suspensions were kept at room temperature for 7 days to allow for any MOF degradation, ion exchange, or surface modifications in each environment. After the 7 day aging period, the UiO-66 solids were recovered from each solution via centrifugation (8000 rpm, 10 min) and thoroughly washed with deionized water to remove any residual salts or media components. The filtered powders were then dried in air at ambient temperature. The dried, aged MOF samples were analyzed by multiple techniques to assess chemical and structural changes resulting from aqueous exposure. FTIR (PerkinElmer Spectrum, USA) and XPS were used to detect changes in functional groups and surface chemistry. Powder XRD (Malvern Panalytical, UK) was performed to determine if any loss of crystallinity or phase changes occurred. TEM (Jeol 1400, Japan) was employed to observe morphological alterations or particle agglomeration. Furthermore, the aged samples were prepared for XAS analysis (Zr K-edge XANES/EXAFS) by loading into Kapton washers (similar to the gas-exposed samples). The samples were measured at ambient atmosphere. This suite of analyses provided a comprehensive assessment of UiO-66 stability and transformation in each aquatic medium. Specific surface areas and pore characteristics of UiO-66 samples were determined by N_2_ adsorption–desorption at 77 K using a volumetric gas sorption analyzer (BELSORP MINI X, Microtrac, Japan). Activated UiO-66 and samples aged for 7 days in ambient air, BHW (simple water) and CCM (complex water) were collected, washed with water and reactivated under dynamic vacuum at 120 °C overnight prior to analysis. BET surface areas were calculated from the adsorption branch in the relative pressure range *P*/*P*_0_ ≈ 0.05–0.25, while total pore volumes were obtained from the adsorbed volume at *P*/*P*_0_ ≈ 0.99; apparent pore diameters were estimated from the adsorption branch using the BJH method.

#### 5.3.3 Tertiary Transformation (*D. magna*)

To investigate MOF transformations under biologically active conditions, an acute exposure of *D. magna* to UiO-66 was conducted in BHW. *D. magna* neonates (5 daphnids/20 mL of BHW) individuals (Bham2 strain, continuous cultured in BHW) were exposed to a dispersion of UiO-66 (preaged in BHW for hierarchical transformation) in BHW at 100 mg/L (≈100 ppm) for 24 h. The exposure was carried out in well-aerated glass vials, with appropriate controls, under constant temperature and photoperiod conditions consistent with standard ecotoxicological protocols,^42^ in the absence of feeding. During this period, the *daphnids* were actively swimming in the suspension, allowing for ingestion of UiO-66 particles. After 24 h, the organisms were removed and gently rinsed with fresh BHW to detach any loosely bound UiO-66 particles. The animals were then lyophilized (freeze-dried) to preserve their internal distribution of metals and to halt any further biochemical reactions prior to analysis. Spatially resolved XRF mapping and micro-XAS measurements were performed on the *D. magna* samples at Diamond Light Source beamline I18 (microfocus spectroscopy beamline). Post exposure to UiO-66 MOFs for 24 h, some of the daphnids were introduced to a fresh BHW medium for depuration. The depurated UiO-66 MOFs were imaged using TEM (JEOL 1400, Japan) to investigate their morphological and structural alterations after passing through the gut of daphnids. Diffraction and composition analysis were performed on the TEM JEOL 2100 and Talos F200X G2 TEM, both operated at 200 kV electron voltage. For TEM, the activated UiO-66 was dispersed onto ultrathin continuous carbon films supported on 200-mesh gold grids. Due to the electron beam-induced damage on the UiO-66,^43^ selected area electron diffraction analysis of the pristine and depurated samples was performed under low-dose exposure. The beam current, measured on the samples, was kept at 0.074 nA by using the lowest condenser aperture (10 μm) and high numerical number of spot size (spot size 6) on both microscopes. For composition analysis, STEM and electron dispersive X-ray spectroscopy (EDS) were performed to produce the distribution map of all presenting elements. STEM-EDS mapping was performed on the Talos F200X G2 TEM with the probe current of 1.2 nA (measured outside the sample), convergent semiangle of 10 mrad, and the dwell time of 100 μs.

## Supplementary Material



## Data Availability

Related biotic transformation data for UiO-66 in *D. magna* were first published in ref 7 and are reused in part in Figure 3 as noted in the caption.

## Notes

Additional data are provided, including physicochemical characterization of UiO-66 (SEM, TEM, PXRD; Figure S1), XPS spectra after exposure to different aqueous media (Figure S2), and compositions of the exposure media (Table S1). Detailed Zr K-edge XAS/XANES and EXAFS analyses under gas-phase and aqueous conditions are presented (Figures S3–S6; Tables S2–S3), along with structural models of the Zr_6_ cluster (Figure S7), comparison of spectra from B18 and I18 beamlines (Figure S8), and EDS spectra of activated versus depurated UiO-66 (Figure S9). Selected *D. magna* XRF/EXAFS data underlying parts of Figure 3 were recently reported by us (ref.^7^) and are reproduced/adapted here (with permission) for completeness and direct comparison with the transformation hierarchy (PDF)
