# Supplementary material for: Mapping the Hierarchical Environmental Transformations of Nanoscale UiO-66 Metal–organic Framework
Source: Environ Sci Technol. 2026 Jan 2;60(2):2122–36. doi: 10.1021/acs.est.5c14487 (PMC12825163; doi:10.1021/acs.est.5c14487)
Supplement: Supplementary file 1 [file es5c14487_si_001.pdf]

Supplementary Information for

Mapping the Hierarchical Environmental Transformations of Nanoscale UiO-66 Metal-organic Framework

Swaroop Chakraborty[a,b]*, Iuliia Mikulska[c]*, Pankti Dhumal[a,b], Nathan Langford[a], Susan Nehzati[c], Rhiannon Boseley[c], Sang Pham[d], Christian Pfrang[a], Manpreet Kaur[a], Eugenia Valsami-Jones[a], Konstantin Ignatyev[c], Dhruv Menon[e], Superb K. Misra[f], Iseult Lynch[a,b]

[a] School of Geography, Earth & Environmental Science, University of Birmingham, Edgbaston, Birmingham, B15 2TT, UK

[b] Centre for Environmental Research and Justice, University of Birmingham, Edgbaston, Birmingham, B15 2TT, UK

[c] Diamond Light Source, Harwell Science and Innovation Campus, Didcot, **OX11 0DE**, UK

[d] Facility of Electron Microscopy, University of Birmingham, Edgbaston, Birmingham, B15 2TT, UK

[e] Department of Chemical Engineering & Biotechnology, University of Cambridge, Cambridge, CB3 0AS UK

[f] Materials Engineering, Indian Institute of Technology Gandhinagar, Palaj, 382355, India

*email(s): s.chakraborty@bham.ac.uk; iuliia.mikulska@diamond.ac.uk

Summary:

- **Number of pages:** 14
- **Number of Figures:** 12 (Figures **S1** – **S12**)
- **Number of Tables:** 4 (Tables **S1** – **S4**)

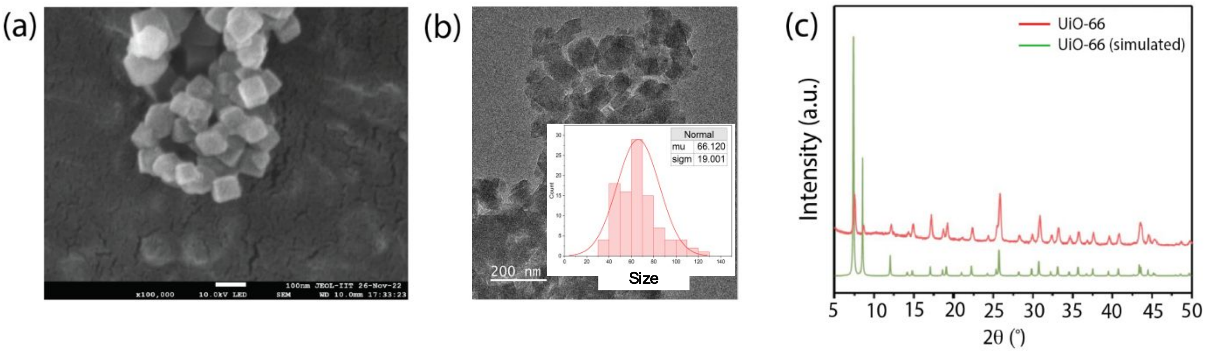

Figure S1: Physicochemical properties of UiO-66 MOFs. (a) SEM image of pristine UiO-66 crystals showing uniform octahedral morphology. (b) TEM micrograph of pristine UiO-66 with size histogram (inset) indicating well-defined crystalline facets and particle connectivity. (c) PXRD patterns of as-synthesised UiO-66 (red) compared with simulated UiO-66 (green), confirming phase purity and crystallinity.

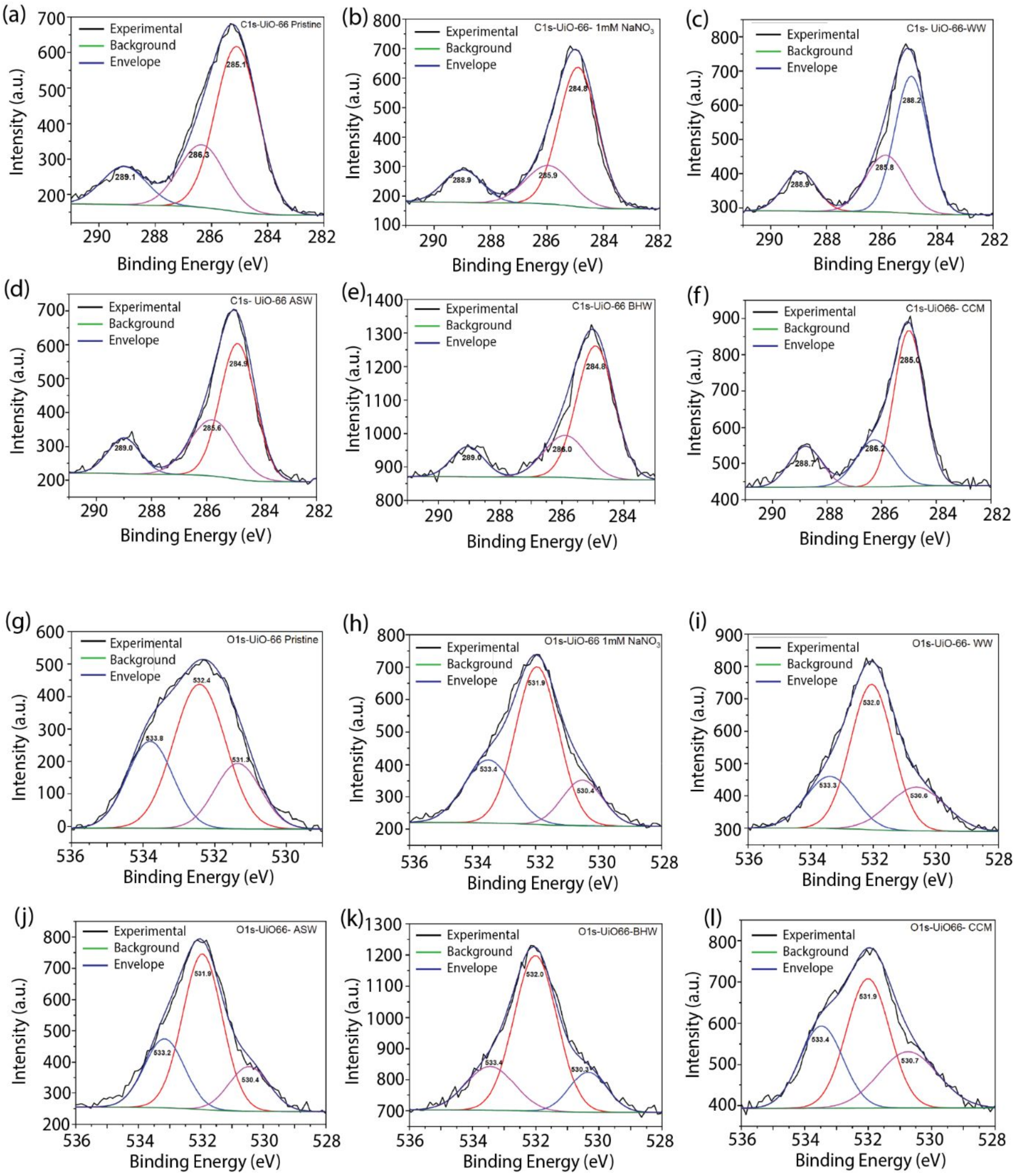

Figure S2. XPS analysis of UiO-66 MOFs exposed to various liquid media for 7 days. (a–f) High-resolution C 1s XPS spectra of UiO-66 after exposure to different media: (a) as-synthesized UiO-66 powder (b) 1 mM $NaNO_3$, (c) WW, (d) ASW, (e) BHW, and (f) CCM containing 10% foetal bovine serum. The composition of each medium is presented in **Table S1**. Peaks correspond to C–C/C–H (~284.8 eV), C–O (~286.3 eV), and O–C=O (~288.9 eV) environments, with notable intensity variations reflecting surface chemistry modifications. (g–l) High-resolution O 1s XPS spectra of UiO-66 in the same media: (g) as-synthesized, (h) 1 mM $NaNO_3$, (i) WW, (j) ASW, (k) BHW, and (l) CCM. Deconvoluted peaks at ~532.4 eV (metal–oxygen), ~531.0 eV (C=O), and ~533.0 eV (adsorbed species/water) reveal changes in oxygen-containing functionalities due to medium-specific interactions.

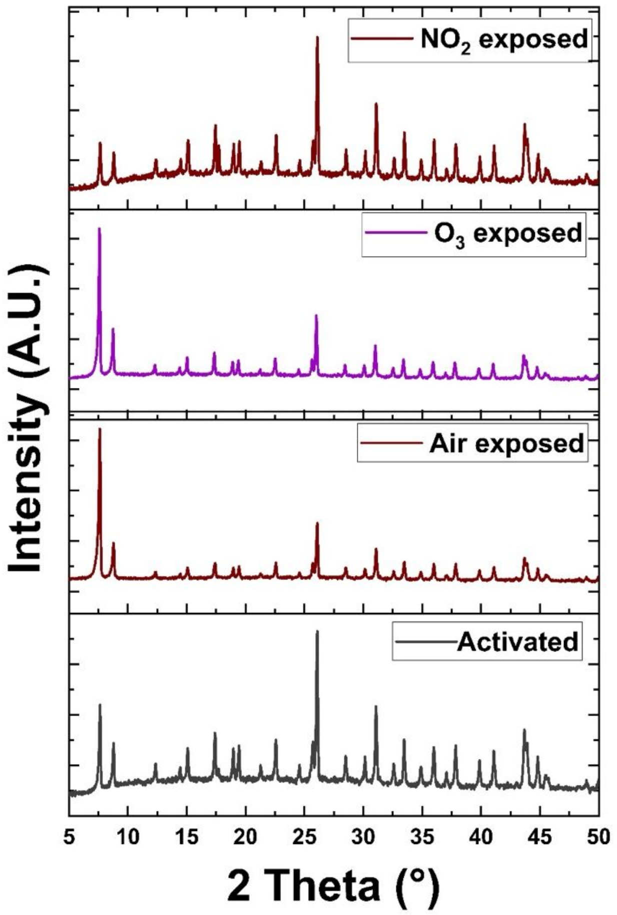

Figure S3. Powder XRD patterns of UiO-66 before and after gas-phase ageing. Activated UiO-66 and samples exposed for 7 days to air, 10 ppm O_3 and 10 ppm NO_2 show identical sets of reflections characteristic of the UiO-66 fcu structure, with no additional peaks indicative of secondary phases or amorphization, confirming retention of bulk crystallinity after oxidative gas exposure.

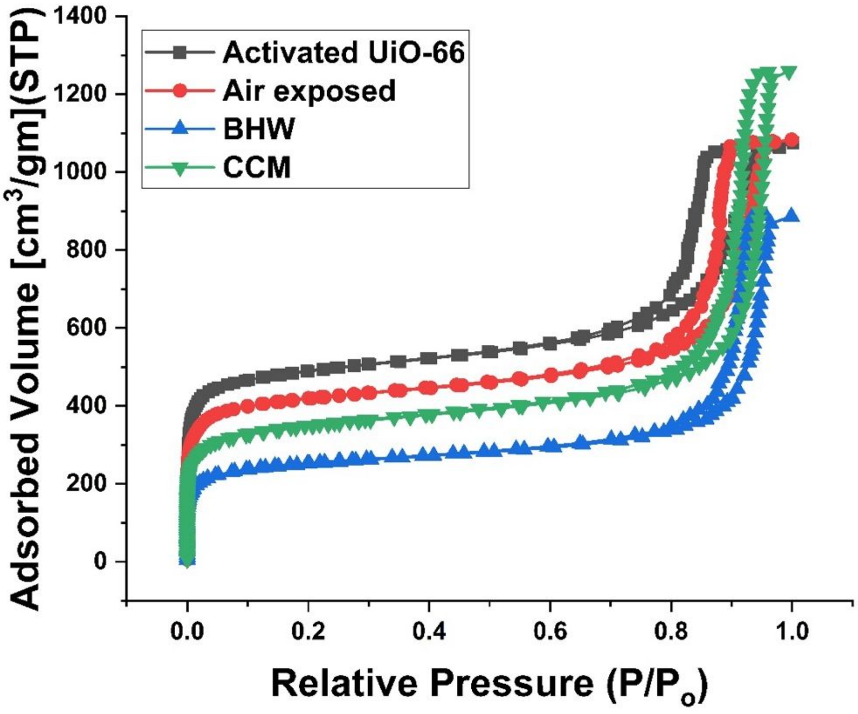

Figure S4: N_2 adsorption-desorption isotherms (77 K) of UiO-66 after different ageing treatments. Isotherms are shown for activated UiO-66 (black squares), air-exposed for 7 days (red circles), borehole-water (BHW)-exposed for 7 days (blue triangles) and cell-culture medium (CCM)-exposed for 7 days (green inverted triangles). The retention of the characteristic microporous profile, with progressive reduction in adsorbed volume, reflects partial loss or blocking of porosity upon ageing, most pronounced for BHW.

Table S1. Composition, properties, and environmental/biological relevance of aqueous media used for UiO-66 exposure experiments. The portion of table discussing the composition of BHW is reproduced from (REFERENCE [1]). Copyright 2025 American Chemical Society.

Medium	Key Components / Composition	Typical pH	Environmental / Biological Relevance	Notes / Source
**1 mM $NaNO_3$**	Sodium nitrate (1 mM) in deionised (DI) water	~7.0	Serves as a low-ionic-strength, chemically simple baseline for studying intrinsic hydrolytic stability without interference from multivalent ions or organics	Minimal complexity; used to identify primary aqueous stability trends of UiO-66
**ASW (Artificial Seawater)**	NaCl (24.60 g); $NaHCO_3$ (0.180 g); KCl (0.670 g); $CaCl_2$ (1.360 g); $MgSO_4.7H_2O$ (6.290 g)	~8.1–8.3	Mimics marine environments: high ionic strength and presence of multivalent cations can promote framework ion exchange, surface charge screening, and agglomeration[2]	Relevant to UiO-66 behaviour in oceanic discharge or marine remediation contexts
**BHW (Borehole Water)**	Ca^{2+} (~60 mg/L), Mg^{2+} (~30 mg/L), Na^+, Cl^-, HCO_3^-, NO_3^- (<10 mg/L), trace metals (Fe, Mn), low Dissolved Organic Carbon	~6.5–7.2	Natural moderately hard groundwater from University of Birmingham borehole; relevant for environmental stability studies in freshwater aquifers and natural exposure scenarios. It is regularly used in our laboratory, and quarterly chemical analyses have confirmed a consistent composition over the past 10 years.	Collected from University of Birmingham borehole on March 2023; low organic content but elevated hardness may stabilise/displace framework ligands
**WW (Simulated Wastewater)**	Milk powder: **150 mg/L** (COD 156 mg/L), Starch: **80 mg/L** (COD 80.6 mg/L) Sodium acetate: **103 mg/L** (COD 39.6 mg/L), Yeast: **24 mg/L** (COD 24 mg/L), Ammonium chloride (NH_4Cl): **21.7 mg/L** (providing 6 mg/L N), Urea: **12.8 mg/L** (providing 6 mg/L N), Potassium dihydrogen phosphate (KH_2PO_4): **13.2 mg/L** (providing 3 mg/L P), Sodium bicarbonate ($NaHCO_3$): **600 mg/L** (providing 300 mg/L $CaCO_3$ alkalinity)	~7.0–7.4	Represents municipal or industrial wastewater[3]; high organic content and nutrients facilitate eco-corona formation and competitive complexation with Zr nodes	Organic-rich matrix may accelerate linker displacement or promote particle flocculation
**CCM (Cell Culture Medium)**	Detailed composition can be found here- https://www.sigmaaldrich.com/GB/en/product/sigma/d6429	~7.2–7.4	Simulates *in vitro* biological exposure; nutrient-rich environment promotes strong bio-corona formation and possible bio-mediated transformations.	Often used for mammalian cell culture; relevant for biomedical MOF applications and *in vitro* toxicity assays

Table S2. Parameters of the nearest neighbours around Zr atoms in the UiO-66 exposed to different gases. N – number of neighbour atoms, R – distance from the Zr atom; σ^2 – Debye-Waller factor. E_0 = 2.3 ± 0.3 eV, S_0^2 = 1.30 ± 0.05, R-factor: 0.0045568.

Zr neighbours	Path parameters	Activated	Air	O_3, 5 ppm, 60 min	O_3, 5 ppm, 120 min	O_3, 10 ppm, 60 min	O_3, 10 ppm, 120 min	NO_2, 10 ppm, 60 min	NO_2, 5ppm, 30 min
O(1)	N	2	2	2	2	2	2	2	2
	R [Å]	2.089 (4)	2.076 (7)	2.088 (4)	2.085 (9)	2.079 (6)	2.074 (5)	2.076 (9)	2.078 (14)
	σ^2 [Å^2]	0.0040 (5)							
O(2)	N	6	6	6	6	6	6	6	6
	R [Å]	2.250 (3)	2.237 (6)	2.249 (3)	2.247 (6)	2.238 (4)	2.238 (4)	2.237 (7)	2.234 (9)
	σ^2 [Å^2]	0.0068 (4)							
O(3)/N(1)	N	-	1	1	1	1	1	1	1
	R [Å]	-	2.396 (16)	-	-	2.397 (9)	2.397 (9)	2.378 (24)	2.378 (24)
	σ^2 [Å^2]	-	0.0017 (8)	-	-	0.0017 (8)		0.0034 (33)	
C	N	4	4	4	4	4	4	4	4
	R [Å]	3.318 (46)	3.296 (70)	3.334 (42)	3.287 (91)	3.298 (67)	3.254 (47)	3.275 (82)	3.301 (130)
	σ^2 [Å^2]	0.0186 (43)							
Zr	N	4	4	4	4	4	4	4	4
	R [Å]	3.525 (4)	3.527 (5)	3.527 (4)	3.520 (9)	3.528 (5)	3.521 (6)	3.522 (9)	3.526 (8)
	σ^2 [Å^2]	0.0057 (4)							

Table S3. Parameters of the nearest neighbours around Zr atoms in the UiO-66 exposed to different liquid media for 7 days. N – number of neighbour atoms, R – distance from the Zr atom; σ^2 – Debye-Waller factor. E_0 = 4.5 ± 0.4 eV, S_0^2 = 1.18 ± 0.04, , R-factor: 0.0068025.

Zr neighbours	Path parameters	Pristine	1mM $NaNO_3$	ASW	WW	BHW	CCM
**O(1)**	**N**	2	2	2	2	2	2
	**R [Å]**	2.092 (4)	2.090 (4)	2.092 (3)	2.092 (3)	2.089 (8)	2.091 (9)
	**σ^2 [Å^2]**	0.0020 (4)					
**O(2)**	**N**	6	6	6	6	6	6
	**R [Å]**	2.245 (4)	2.243 (4)	2.244 (3)	2.243 (3)	2.242 (6)	2.241 (7)
	**σ^2 [Å^2]**	0.0049 (4)					
**O(3)/N(1)**	**N**	1	1	1	1	1	1
	**R [Å]**	2.402 (6)	2.402 (6)	2.402 (6)	2.402 (6)	2.402 (6)	2.402 (6)
	**σ^2 [Å^2]**	0.0010 (5)					
**C**	**N**	4	4	4	4	4	4
	**R [Å]**	3.152 (31)	3.147 (32)	3.141 (23)	3.142 (21)	3.163 (84)	3.152 (99)
	**σ^2 [Å^2]**	0.0166 (52)					
**Zr**	**N**	4	4	4	4	4	4
	**R [Å]**	3.542 (3)	3.538 (4)	3.539 (2)	3.540 (3)	3.537 (10)	3.540 (17)
	**σ^2 [Å^2]**	0.0074 (3)					

Table S4: Textural properties of UiO-66 after gas-phase and aqueous ageing, derived from N_2 adsorption–desorption at 77 K. BET surface area, monolayer capacity (Vm), BJH pore diameter and total pore volume are reported for activated UiO-66 and for samples aged for 7 days in air, BHW and CCM, corresponding to the isotherms shown in **Figure S4**.

Sample	Surface area (m^2/g)	Vm (cm^3/g)	Pore diameter (nm)	Pore volume (cm^3/g)
**Activated UiO-66**	1606.5	369.11	2.0596	1.6544
**CCM**	1375	315.92	2.4324	1.6723
**AIR**	1149.5	264.11	3.3796	1.9425
**BHW**	831.8	191.11	3.2798	1.3641

Zr K-edge XAS analysis

Qualitative comparison of UiO-66 samples: activated, and those exposed to air, natural borehole water, and Daphnia magna with Zr reference compounds.

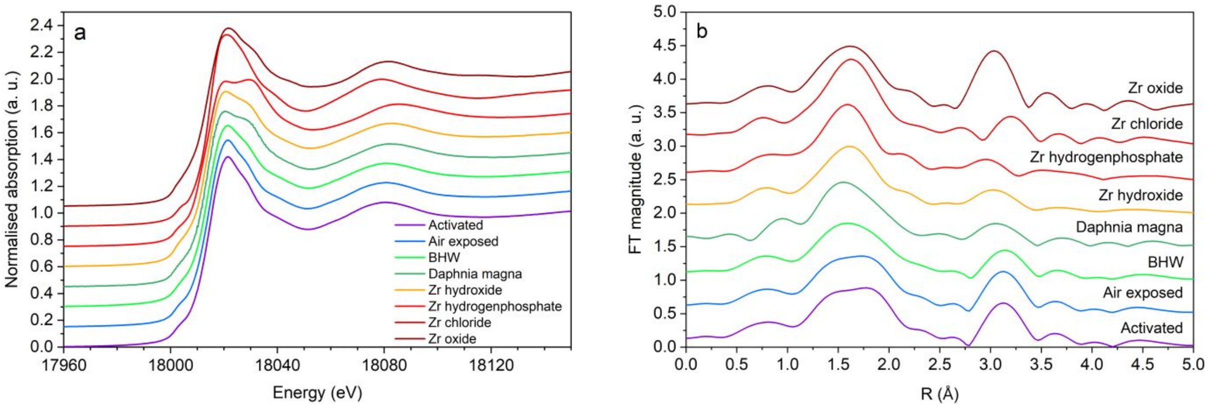

Figure S5. Comparison of Zr K-edge spectra: (a) XANES and (b) Fourier-transformed EXAFS for activated UiO-66, samples exposed to air, BHW, and *D. magna*, alongside zirconium reference compounds (Zr hydroxide, Zr hydrogen phosphate, Zr chloride, and Zr oxide).

Qualitative comparison of as synthesized and activated UiO-66 samples.

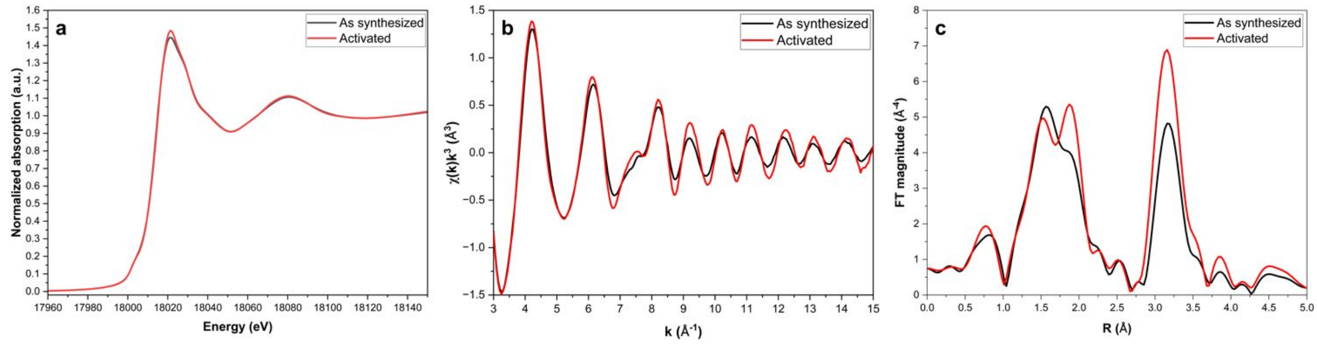

Figure S6. Comparison of Zr K-edge (**a**) XANES, (**b**) EXAFS spectra, and (**c**) Fourier transform magnitudes of EXAFS spectra for as-synthesized and activated UiO-66 samples. The as-synthesized UiO-66 were vacuum dried overnight at 120 °C for activation. The measurements were performed at B18 beamline at Diamond Light Source, UK.

Quantitative EXAFS analysis.

Quantitative Zr K-edge EXAFS analysis has been performed on as-synthesized and activated UiO-66 samples exposed to different air, reactive gases and aqueous media. The spectral analysis program FEFF[4] was used to construct a model comprised of all single and significant multiple scattering paths of the photoelectron up to 3.7 Å. For each Zr neighbour shell the distance (R) and Debye-Waller factor (s^2) were allowed to vary in the fit, while number of neighbours (N) at each distance was fixed to the values defined in the crystallographic data (CIF file Information card for entry **837796** [5]). A common variable linear expansion coefficient was used in all multiple scattering paths to restrain the variation of neighbour distance. In

addition, a common shift of energy origin (ΔE_0) for all scattering paths was allowed to vary in the fit. EXAFS amplitude reduction factor (S_0^2) was allowed to vary as well.

A very good agreement between the model and experimental spectra of activated and exposed to different media UiO-66 samples was found in the R-range 1.3 – 3.7 Å and in the k-interval of [3.7 $Å^{-1}$ – 14.5 $Å^{-1}$] using k^3-weight **(Figure S5)**. The fitting of the activated sample and all gas-exposed samples was performed separately from the fitting of the activated sample and those exposed to liquid media. Due to limitations in Artemis software - specifically, the maximum number of spectra and scattering paths that can be simultaneously imported into a project file—it was not possible to fit all the spectra together. In this instance, simultaneous relaxation was applied in the fitting process, with certain parameters of the fitted spectra constrained to common values. Specifically, the Debye-Waller factors for the corresponding paths were constrained to the same values across all the samples. A common shift of energy origin (ΔE_0) and a common value of amplitude reduction factor (S_0^2) were used for all the samples. The amplitude reduction factor typically ranges from 0.7 to 0.9. However, due to the inherent uncertainty in EXAFS, the S_0^2 value might occasionally exceed one in practical scenarios. Similar values of amplitude reduction factor, which exceed one, have been observed in other XAS studies on the UiO-66 system [5,6]. The results of preliminary EXAFS fit for all the samples exposed to gaseous and aquatic media are shown in **Tables S2 and S3** respectively. The results show that the local structure around Zr atoms is very similar in all the samples. Including an additional Zr–O(3) scattering path at 2.4 Å significantly improved the EXAFS fit for samples exposed to all aquatic media, air, NO_2, and 10 ppm O_3. The impact on fit quality is illustrated in **Figure S6**.

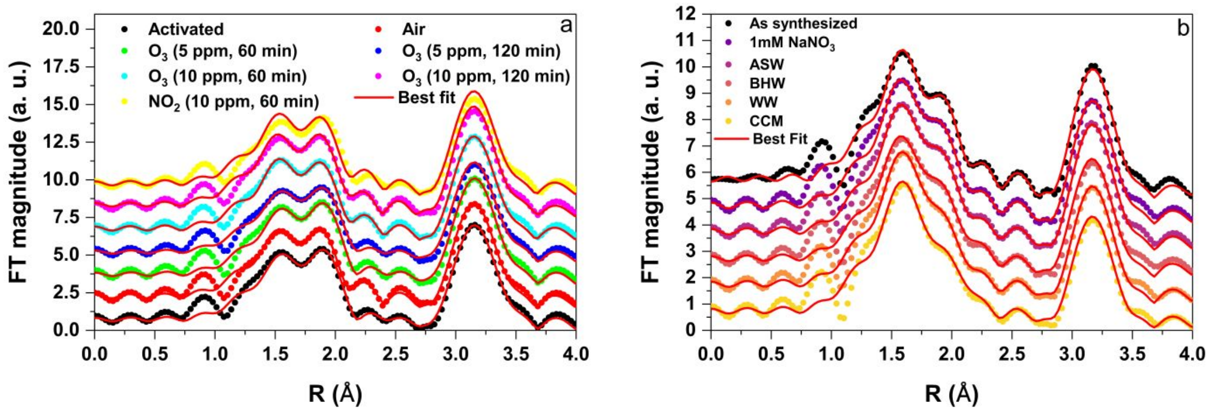

Figure S7. XAS Analysis of UiO-66 under Environmental Exposure. Fourier-transformed Zr K-edge EXAFS spectra measured on UiO-66 samples exposed to (a) gas-phase and (b) aqueous environments. Experimental data (dots) are overlaid with best-fit models (red lines). Spectra are vertically offset for clarity.

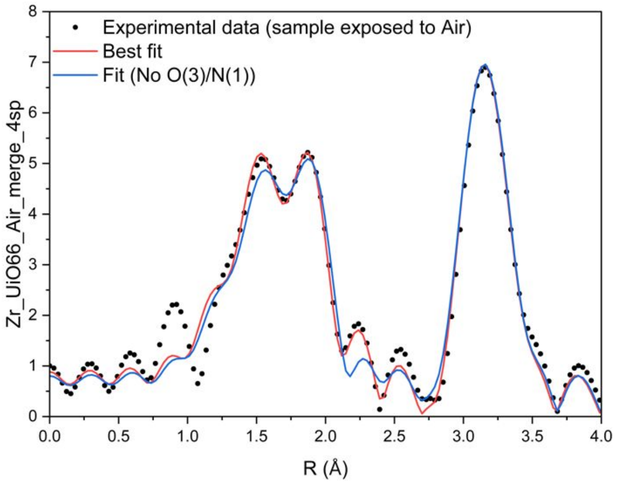

Figure S8. Fit Comparison of Zr K-edge EXAFS Spectra. Fourier-transformed EXAFS spectra of air-exposed UiO-66 (black dots) compared with best-fit models, including (R-factor: 0.00613) and excluding (R-factor: 0.01843) the Zr–O(3)/N(1) single scattering path. Spectra are shown for clarity of fit differences.

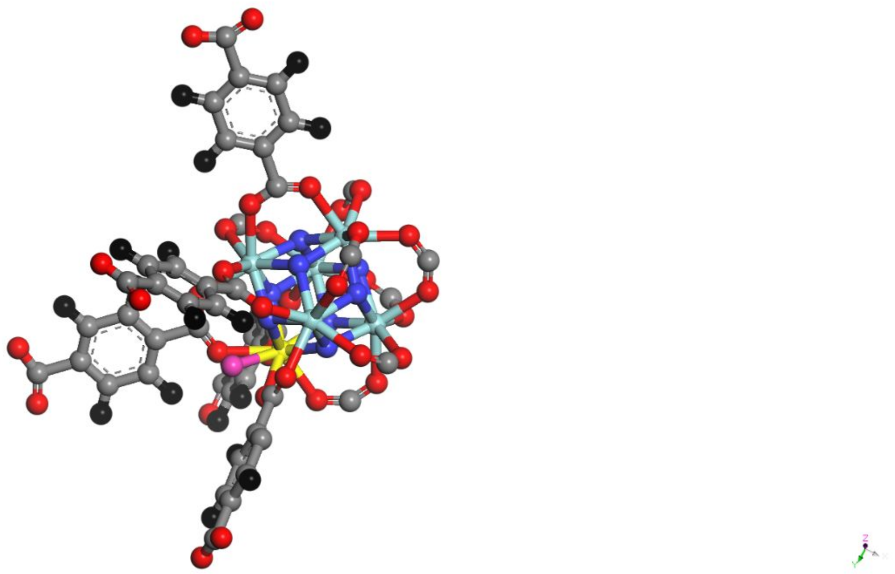

Figure S9. Structural Representation of the Zr6 cluster in UiO-66 MOF. The structures display the Zr_6 cluster core along with the essential portion of the organic linkers. The majority of the organic linkers were removed for clarity. Blue spheres – oxygen atoms O(1), red speres – oxygen atoms O(2), magenta sphere – oxygen atoms absorbed within the pores O(3), cyan spheres – zirconium atoms, yellow – represents the zirconium absorbing atom, grey spheres – carbon atoms, black speres – hydrogen atoms**.**

Comparison of Zr K-edge spectra measured on I18 and B18 beamlines at Diamond light source, UK

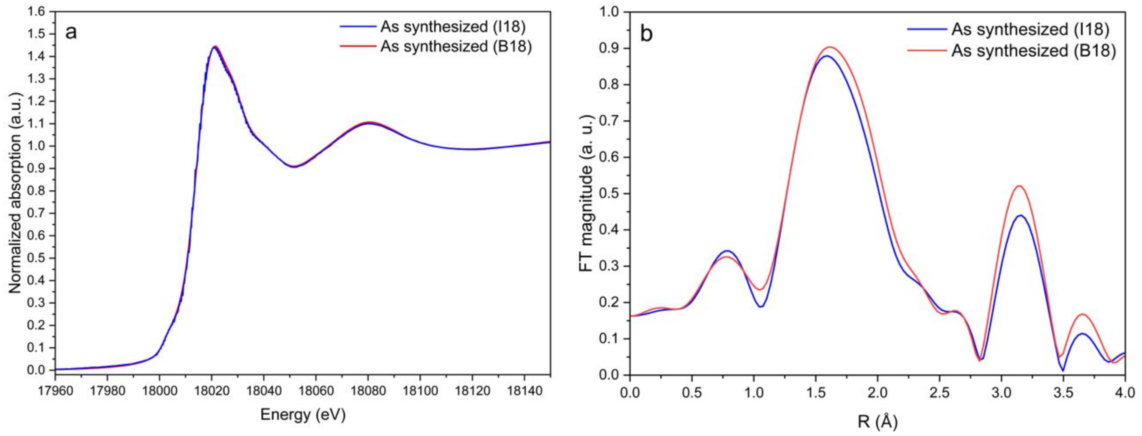

Figure S10. Comparison of Zr K-edge Spectra from XANES and EXAFS. Zr K-edge (a) XANES and (b) Fourier-transformed EXAFS magnitudes for as-synthesized UiO-66, measured at the I18 and B18 beamlines, respectively. The figure is reproduced from (REFERENCE [1]). Copyright 2025 American Chemical Society.

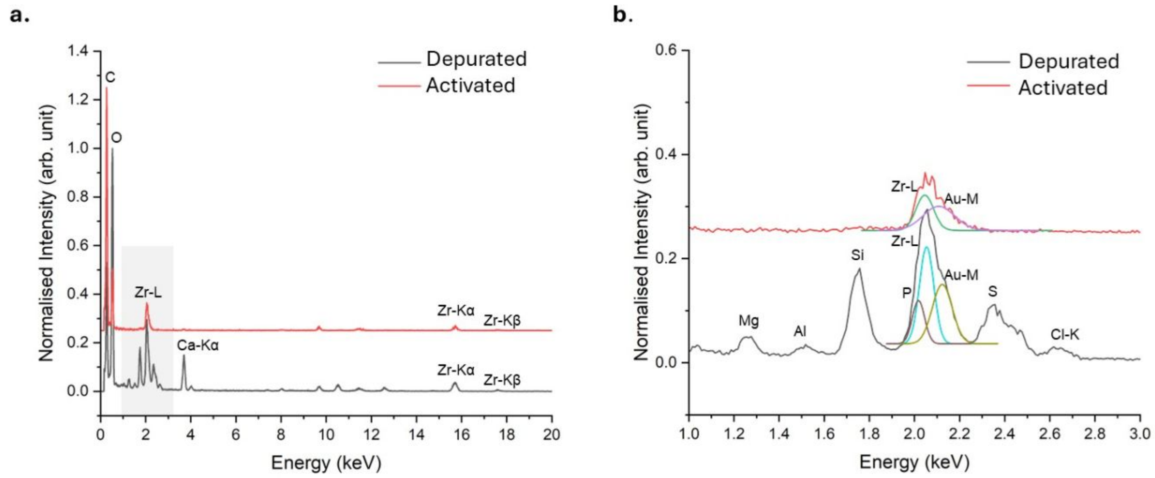

Figure S11. EDS spectra of activated and depurated UiO-66 particles. (a) Overview spectra showing the presence of Zr in both samples. The depurated sample exhibits a marked increase in the O signal relative to C along with several additional peaks in the 1–3 keV range, in comparison to the as-synthesised sample. (b) Magnified view of the 1–3 keV region, highlighting the presence of other metallic elements as well as S and Cl. Peak fitting of the ~2 keV feature reveals the presence of P in the depurated sample, suggesting the formation of Zr-phosphate species compared with the activated material.

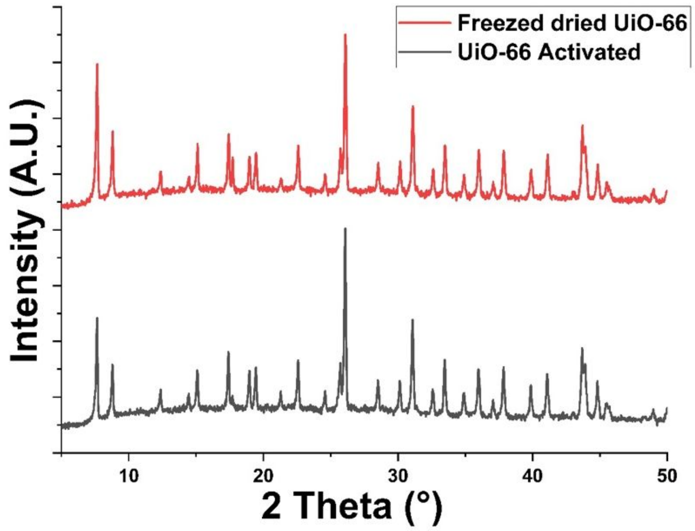

Figure S12. Powder XRD patterns of UiO-66 before and after borehole-water exposure and freeze-drying. The activated UiO-66 sample (black) and the UiO-66 sample exposed to BHW for 7 days and subsequently freeze-dried at –55 °C (red) exhibit identical sets of reflections characteristic of the UiO-66 fcu structure, with no additional peaks or loss of crystallinity, indicating that the freeze-drying procedure does not induce detectable framework degradation.

References

1. Chakraborty, S. *et al.* Biotic Transformation of Abiotically Stable Nanoscale UiO-66 Metal–Organic Framework by Daphnia magna Results in Chronic Reproductive Toxicity. *ACS Nano* https://doi.org/10.1021/ACSNANO.5C16532 (2025) doi:10.1021/ACSNANO.5C16532.
2. of Science, O. Methods for Measuring the Acute Toxicity of Effluents and Receiving Waters to Freshwater and Marine Organisms Fifth Edition. (2002).
3. Kayranli, B. & Ugurlu, A. Effects of temperature and biomass concentration on the performance of anaerobic sequencing batch reactor treating low strength wastewater. *Desalination* 278, 77–83 (2011).
4. Rehr, J. J. *et al.* Ab initio theory and calculations of X-ray spectra. *C R Phys* 10, 548–559 (2009).
5. Valenzano, L. *et al.* Disclosing the complex structure of UiO-66 metal organic framework: A synergic combination of experiment and theory. *Chemistry of Materials* 23, 1700–1718 (2011).
6. Ronda-Lloret, M. *et al.* Mixed-Valence Ce/Zr Metal-Organic Frameworks: Controlling the Oxidation State of Cerium in One-Pot Synthesis Approach. *Adv Funct Mater* 31, 2102582 (2021).
